# Identification of novel interferon responsive protein partners of human leukocyte antigen A (HLA-A) using cross-linking mass spectrometry (CLMS) approach

**DOI:** 10.1038/s41598-022-21393-z

**Published:** 2022-11-12

**Authors:** Ashita Singh, Monikaben Padariya, Jakub Faktor, Sachin Kote, Sara Mikac, Alicja Dziadosz, Tak W. Lam, Jack Brydon, Martin A. Wear, Kathryn L. Ball, Ted Hupp, Alicja Sznarkowska, Borek Vojtesek, Umesh Kalathiya

**Affiliations:** 1 Institute of Genetics and Cancer, University of Edinburgh, Edinburgh, EH4 2XR Scotland, UK; 2 Department of Experimental Biology, Faculty of Science, Masaryk University, Kamenice 5, 625 00 Brno, Czech Republic; 3 International Centre for Cancer Vaccine Science, University of Gdansk, ul. Kładki 24, 80-822 Gdansk, Poland; 4 RECAMO, Masaryk Memorial Cancer Institute, Zlutykopec 7, 65653 Brno, Czech Republic; 5 School of Biological Sciences, Institute of Structural and Molecular Biology, University of Edinburgh, Edinburgh, EH9 3JR UK

**Keywords:** Biological techniques, Biophysics, Computational biology and bioinformatics, Molecular biology, Structural biology, Molecular medicine, Physics

## Abstract

The interferon signalling system elicits a robust cytokine response against a wide range of environmental pathogenic and internal pathological signals, leading to induction of a subset of interferon-induced proteins. We applied DSS (disuccinimidyl suberate) mediated cross-linking mass spectrometry (CLMS) to capture novel protein–protein interactions within the realm of interferon induced proteins. In addition to the expected interferon-induced proteins, we identified novel inter- and intra-molecular cross-linked adducts for the canonical interferon induced proteins, such as MX1, USP18, OAS3, and STAT1. We focused on orthogonal validation of a cohort of novel interferon-induced protein networks formed by the HLA-A protein (H2BFS-HLA-A-HMGA1) using co-immunoprecipitation assay, and further investigated them by molecular dynamics simulation. Conformational dynamics of the simulated protein complexes revealed several interaction sites that mirrored the interactions identified in the CLMS findings. Together, we showcase a proof-of-principle CLMS study to identify novel interferon-induced signaling complexes and anticipate broader use of CLMS to identify novel protein interaction dynamics within the tumour microenvironment.

## Introduction

Prior to the onset of the adaptive immune response, the innate host defense system establishes an antimicrobial response, which is mediated by a family of secreted α-helical cytokines known as interferons (IFN). Type I-IFN species IFNα and IFNβ activate cellular responses, including antiviral, pro-apoptotic, pro-inflammatory, and anti-proliferative states. There are 13 IFNα subtypes known in humans, all of which are clustered on chromosome 9^1^. Surprisingly, only IFNα2 has been explored for use in clinical settings. Lately, emphasis on the study of additional IFNα subtypes has been highlighted. A recent study identified IFNα14 as one of the most effective subtypes for restricting the replication of both hepatitis B virus^2^ and HIV-1^3,4^ when compared to the canonical subtype IFNα2.

It has been well established that activated type I-IFN receptor complex (IFNAR1 and IFNAR2) triggers a signal transduction cascade mediated by Janus kinases TYK2 and JAK1^5,6^. These Janus kinases phosphorylate signal transducers and activators of transcription proteins (STAT1 and STAT2) on tyrosine residues to initiate SH2 domain-mediated heterodimerization^6^. Subsequently, IRF9 binds the STAT heterodimer to form a trimeric IFN-stimulated gene factor 3 (ISGF3) complex that translocates to the nucleus, inducing transcription of over 2000 interferon-stimulated genes (ISGs)^5–8^.

The ISGs form the backbone of the innate immune system, particularly in response to viral attack. As a first line of defense against viral infections, cells rapidly deploy an extensive interaction of cellular proteins possessing a wide range of biological activities. Among these proteins are pattern-recognition receptors, signalling molecules, transcription factors and proteins with direct antiviral functions along with negative regulators of the immune response^9^. Most of the knowledge on ISG activities has been gained from functional screens, either using overexpression screens^10,11^ or gene suppression techniques (siRNA, RNAi, and CRISPR)^12,13^ in which single ISGs are expressed or repressed, and their activity is tested against different viruses. Even though these studies identified antiviral properties for individual ISGs, underlying molecular mechanisms behind each one largely remain unknown. It is widely accepted that many proteins interact with one or more cellular factors to achieve full activity; hence, either ISGs interact directly with each other, or their interactions are mediated by cellular proteins. For instance, a recent study using photo-crosslinking proteomics identified an ATPase, VCP/p97 as the primary interaction partner of IFITM3 and its inhibition leads to defective IFITM3 lysosomal sorting, turnover, and co-trafficking with virus particles^14^. Using immunoprecipitation we have identified VAPA, a vesicle associated protein, as an interaction partner of IFITM1/2/3, which mediates cholesterol-mediated viral maturation and this has been supported by another study using yeast two-hybrid system^15,16^.

A fundamental biological process involved in the suppression of infection and malignant transformation is antigen presentation, which is mediated by major histocompatibility complex (MHC) molecules. Peptides (8–12 amino acids long) originating from the degraded proteins, prematurely terminated or misfolded proteins are loaded on to the MHC-I heterodimer (comprised of MHC-I heavy chain and light chain named β-2-microglobulin; β2M)^17,18^. The resulting stable MHC-I trimers are transported to the cell surface to present an intracellular peptide to CD8+ T cells (cytotoxic T cells)^17^. T cells recognize and eliminate these pathogen and tumor-specific antigen bearing cells. As in consequence, pathogens and tumor cells often downregulate the antigen presentation process to evade immune surveillance. Moreover, MHC-I is downregulated in 40–90% of human tumors and often correlates with a worse prognosis^19^.

Genes involved in response to pathogens are required to rapidly switch between resting-state and active transcription state. Therefore, several cellular proteins are assumed to be involved in coping with the high demand of IFNs within a short timeframe, including remodeling and modification of promoter chromatin^20,21^. Most studies have focused on identification of protein partners of individual ISGs in the presence of IFN(s). Several proteomics and transcriptomic studies on model cell systems have illuminated the impact of IFNs on the cellular landscape. Yet, despite growing knowledge on the dynamic changes induced by IFNs, we still know relatively little about the engagement of ISGs. When considering the complexity and time-dependent dynamics of the interferon signalling, two questions arise: (i) if multiprotein complexes involved in rapid signalling can be stabilized and captured, and (ii) could these interactions be mapped in three-dimensional space?

To address these questions, we implemented disuccinimidyl suberate (DSS)-mediated chemical cross-linking combined with mass spectrometry (CLMS) to investigate IFNα-induced protein interaction networks and their dynamics. The DSS adds covalent bonds between proximal residues of proteins and/or protein complexes in vivo. Subsequent MS analysis reveals the specific cross-linking sites, which reflect spatial proximity of regions within a particular protein, called intra-links, or of subunits in a protein complex, called inter-links. Using this approach, we have identified several novel protein–protein complexes as well as multiprotein interaction networks induced by interferon exposure. By further validation of a subset of these novel interactions, we demonstrate H2BFS (histone H2B type F-S; hereafter mentioned as H2B) and MDN1 as binding partners of HLA-A.

## Results

### In-situ CLMS to study interferon stimulated proteins

**Figure 1:**
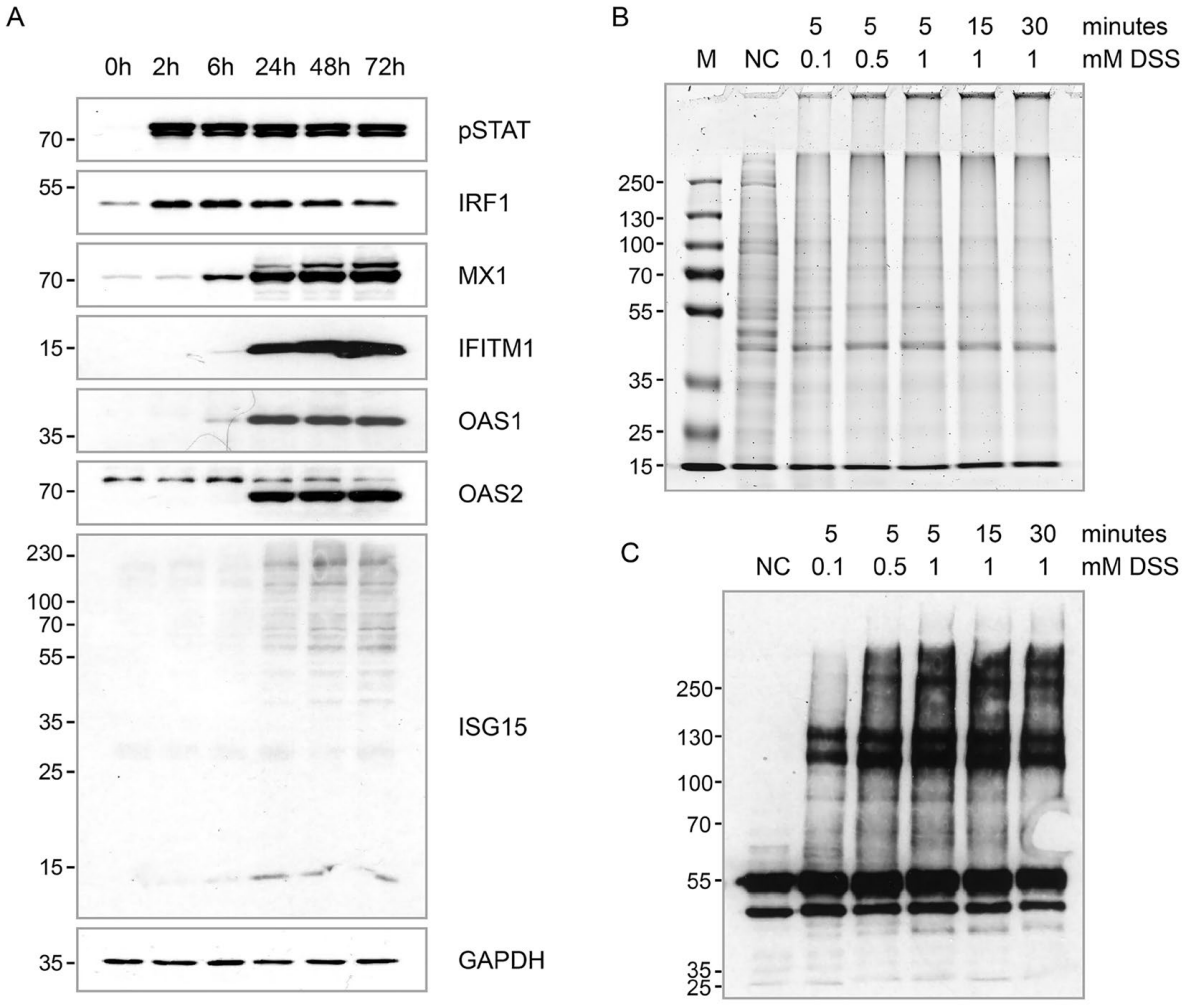
Differential protein expression response in Flo-1 cells following IFNα treatment. **(A)** Analysis of protein expression in Flo-1 cells following treatment with 10 ng/ml IFNα for 2, 6, 24, 48, and 72 h by immunoblotting using the indicated antibodies against ISGs. **(B)** Coomassie blue stained SDS-PAGE gel of whole cell extract after cross-linking with DSS for indicated time and concentration. **(C)** Representative immunoblot probed with p53 (DO-1) antibody of the same samples to assess the degree of protein cross-linking.

Flo-1 cells are one of the most established in vitro Esophageal Adenocarcinoma models as they recapitulate key characteristics of tumours in the esophageal tube^22,23^. However, not all tumours are immunogenic, and to determine if Flo-1 cells show response to interferon treatment, we treated Flo-1 cells with 10 ng/ml IFNα for up to 72 h. Flo-1 cells showed early induction of pSTAT1 and IRF1 starting at 2 h after treatment, which was sustained over the 72 h time-course with a time-dependent decrease in IRF1 steady state levels (Fig. 1A). The ISGs (MX1, IFITM1, OAS1/2, and ISG15) were found to be highly induced after 6 h, mimicing classic intermediate and late responses to IFNα (Fig. 1A). These data together indicate that this cell model can be used to study the interferon response.

To capture the protein interaction landscape in-situ, we used DSS, a widely used cross-linking reagent, for its high membrane permeability and relatively short reaction time. The short reaction time helps to prevent formation of the large, cross-linked protein aggregates thereby maintaining the stability of the cross-linker. To determine the optimum concentration of the DSS and to avoid over cross-linking, we first treated the cells with 5, 2.5 and 1 mM DSS for 5, 10, 5 and 30 min each, and analysed the lysates by Coomassie staining SDS-PAGE (data not shown). The cell lysate appeared to be highly cross-linked at the lowest concentration and shortest time point. DSS was therefore titrated to 1, 0.5 and 0.1 mM for 5 min (Fig. 1B). Optimal cross-linking was observed with 0.5 mM DSS for 5 min and these conditions were selected for IFNα treated cells. Additionally, Fig. 1C represents an immunoblot probed with p53 (DO-1) antibody to assess the degree of protein cross-linking.

**Figure 2:**
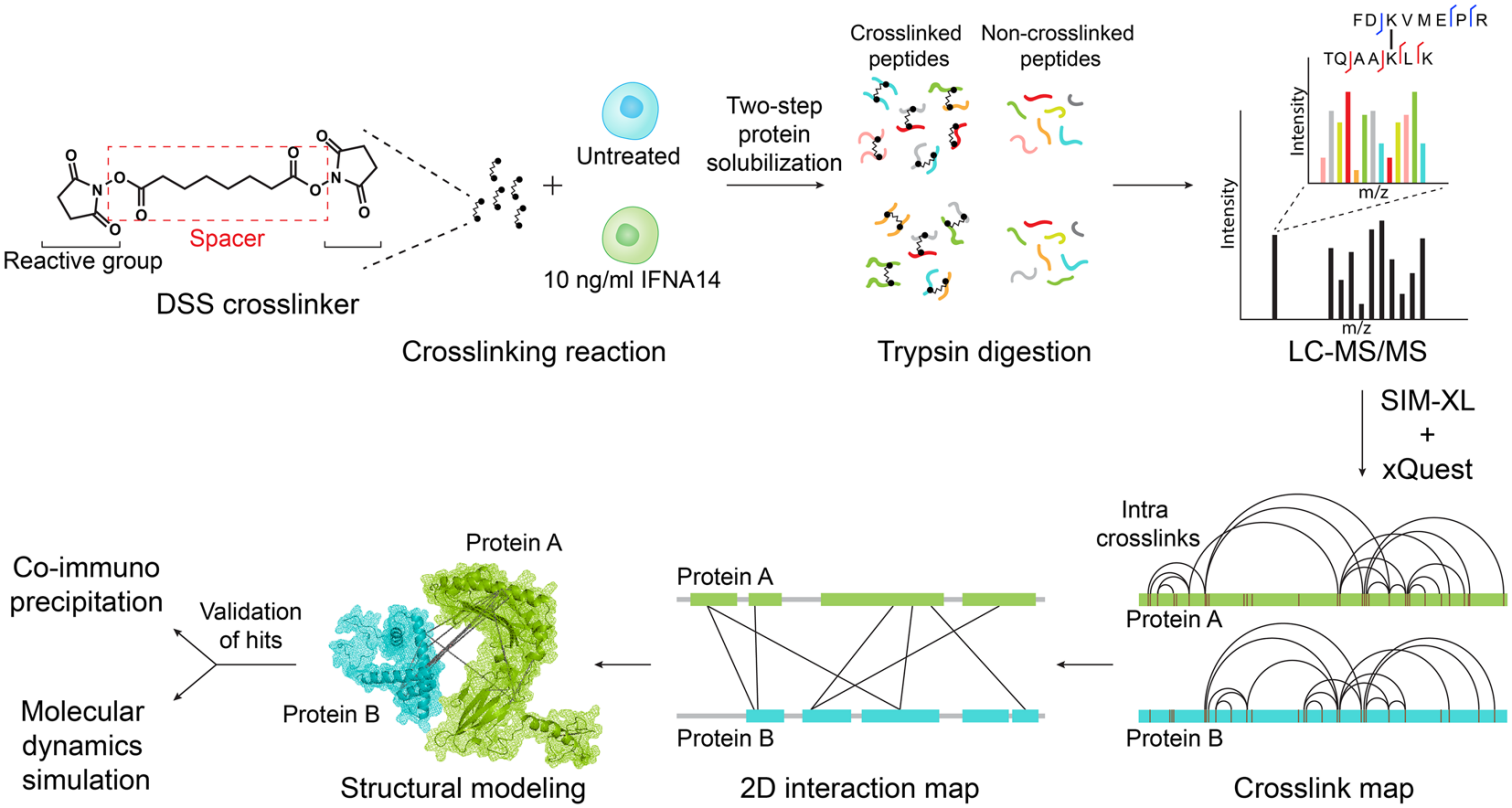
Schematic overview of the CLMS approach. Flo-1 cells were treated with 10 ng/ml of IFNα for 24 h and the proteins were cross-linked in-situ using DSS followed by cell lysis and trypsin digestion. The cross-linked samples were analyzed using an Orbitrap mass spectrometer and peptide precursors were further selected for fragmentation during LC–MS/MS. Two linked peptides were identified from the obtained spectra using the Spectrum Identification Machine for Cross-Linked Peptides (SIM-XL) program and all the linkages were merged into a complex network using computational pipelines. Low confidence interactions were filtered out based on false discovery rate (FDR) estimation. A few of the novel high-confidence protein–protein interactions were further validated using co-immunoprecipitation, and the conformational changes of the complex were studied using molecular dynamics (MD) simulation.

The Flo-1 cells were treated with 10 ng/ml IFNα for 24 h prior to cross-linker addition. Cross-linked cells were subsequently lysed using a two-step protein solubilization method and proteins were processed by the FASP method (Fig. 2)^24,25^. Cross-linked tryptic peptides were analyzed by mass spectrometry (Fig. 2). Next, the MS/MS spectra were aligned to protein sequences and quantitative analysis was carried out using MaxQuant^26,27^. The cross-linked peptides were identified from the obtained spectra using the SIM-XL program and individual linkages were merged into a complex network using an open-source computational software pipeline xQuest^28^ along with SIM-XL^29^ (Fig. 2). SIM-XL identifies protein–protein interaction, intra-links and mono-links in either simple or complex protein mixtures and provides scripts to visualize the interactions in the protein structure. In addition, it ranks each cross-link as an ID score depending on the quality of the MS/MS spectra^29^. Several high-confidence protein–protein interactions and complexes were identified, and a cohort of the novel interactions were further investigated using co-immunoprecipitation and the conformational changes of the complexes were studied using molecular dynamics (MD) simulation (Fig. 2)^30,31^.

### Identification of cross-linked interferon induced proteins

**Figure 3:**
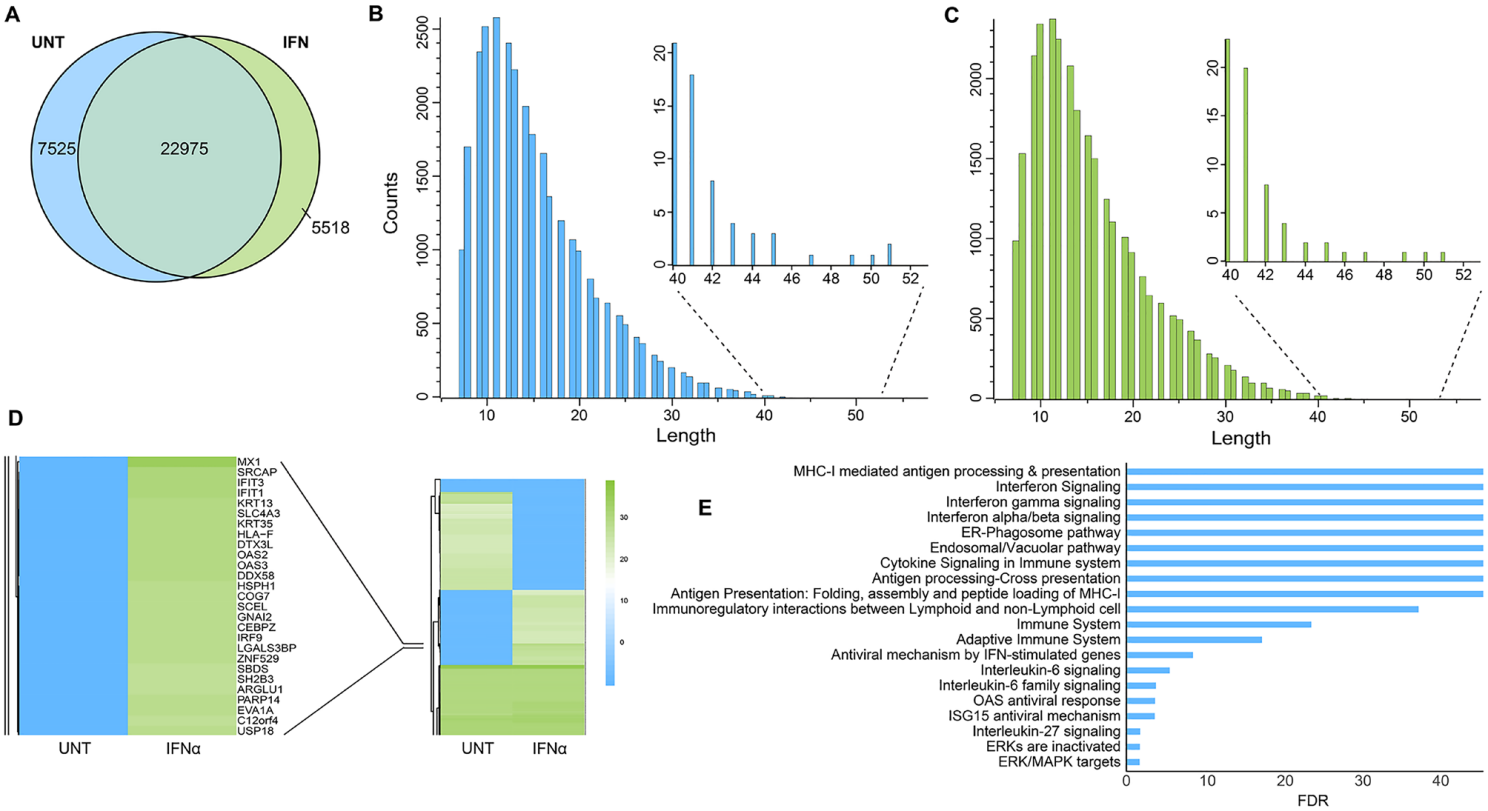
Identification of differentially expressed cross-linked proteins in response to IFNα (data retrieved from MaxQuant). **(A)** Venn diagram representing common and exclusive peptide counts identified in the IFNα14 treated and untreated Flo-1 samples. Peptide length distribution from the untreated **(B)** and IFNα **(C)** treated cross-linked samples. **(D)** Heatmap representing log2(LFQ intensities) between untreated and IFNα14 treated Flo-1 cells. The left panel depicts the most upregulated proteins in presence of IFNα. **(E)** A bar plot representing top 20 enriched pathways following IFNα treatment. IFNα responsive upregulated proteins over fourfold change were analyzed by the Reactome pathway database.

A total of ~ 30,500 and ~ 28,500 peptides were detected in the unstimulated and IFNα stimulated samples, respectively (Supplementary Table S1, Fig. 3A) using MaxQuant. Peptide length distribution for both conditions showed a higher proportion of larger peptides that suggests the presence of cross-linked peptides (Fig. 3B,C). Moreover, in the IFNα treated samples, a higher proportion of larger peptides were present in the range of 40–55 (Fig. 3C). Mapping proteins, against log2 intensities, showed classic interferon-stimulated proteins as the most enriched compared to untreated samples, this included MX1, IFIT1/3, OAS2/3, DDX58, and HLA-F (Fig. 3D). Pathway analysis of proteins that were enriched more than threefold in response to IFNα treatment using Reactome pathway database showed MHC-I mediated antigen presentation and processing as the most dominant pathway (Fig. 3E). Consistent with earlier reports, OAS and ISG15 mediated antiviral response as well as IFNα/β and cytokine signalling were among the upregulated pathways. Further, lysine and serine specific cross-links of proteins were identified from the initially obtained MS/MS spectra using SIM-XL. A recent study has reported 104 ISGs by conducting a meta-analysis of single ISG overexpression studies performed in 5 cell types, covering 20 viruses from 9 virus classes^9^. However, to overcome the computational limitation of screening a big dataset, we started with a smaller dataset and explored possible interactions between the IRDS gene list reported in Padariya et al*.*^28^ out of which, the majority are ISGs.

### Identification of a novel interferon-stimulated protein network based on in-situ cross-link

**Figure 4:**
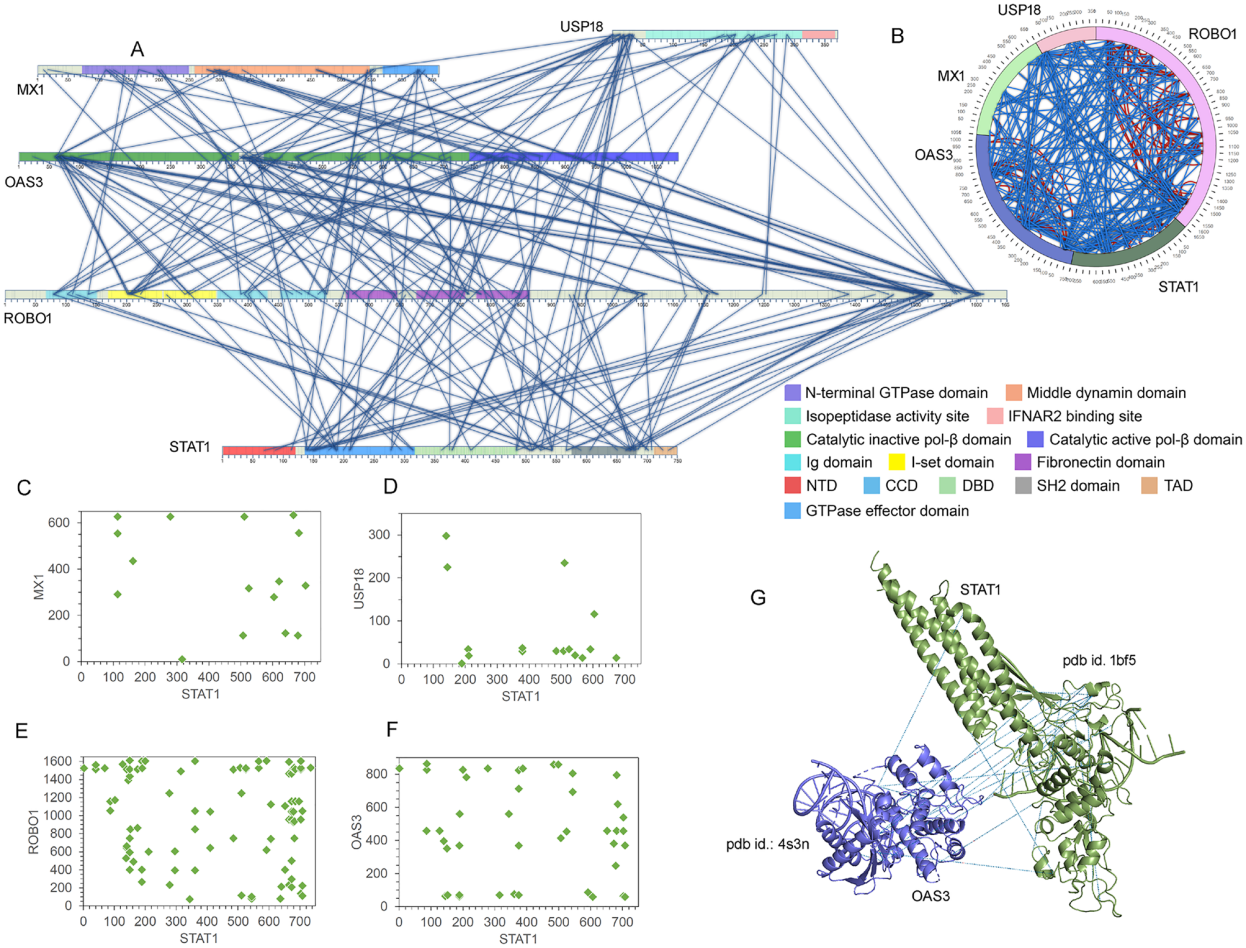
Protein–protein intermolecular network of ISGs identified in the IFNα treated cross-linked cells. **(A)** 2D interactive map (generated in SIM-XL program^29^) showing the protein–protein interactions, the lines represent intermolecular interactions (cross-link score cut-off was set to 3.5). Domains of different identities are labeled with their respective colors^32^: MX1 domains; Dynamin_N (73–249), Dynamin_M (259–547), and GED (569–660). OAS3 domains; OAS1_C (160–344), OAS1_C (559–745), NTP_transf_2 (780–872), and OAS1_C (903–108). ROBO1 domains; Ig_3 (67–151), I-set (170–258), I-set (262–347), Ig_3 (350–432), Ig_3 (454–529), fn3 (562–646), fn3 (678–758), and fn3 (777–864). STAT1 domains; STAT_int (2–120), STAT_alpha (143–309), STAT_bind (321–458), SH2 (573–657), and STAT1_TAZ2bind (715–739). **(B)** The circular viewer of the cross-linked proteins (MX1, UBP18, OAS3, ROBO1, and STAT1), with identified inter- and intra-interactions colored in blue and red, respectively. The cross-link score cut-off was set to 3.5. Dot plots representing the interacting sites of STAT1 with MX1 **(C)**, USP18 **(D)**, ROBO1 **(E)** and OAS3 **(F)**, with interaction site K or S between two peptides. In the plots, the cross-link score cut-off was set to 3.0. **(G)** Different interactions sites between STAT1 and DI domain of OAS3 overlaid over their protein structure in the PyMol (The PyMOL Molecular Graphics System, Version 2.0 Schrödinger, LLC.); STAT1 (pdb id.: 1bf5^33^) and OAS3 (pdb id.: 4s3n^34^) program.

Interferon-mediated stimulation of ISGs is well documented, but at the molecular level how these proteins culminate in a wide range of biological functions is poorly understood. We looked at the high-confidence protein interactions between known ISGs. Interestingly, we identified a network involving MX1, USP18, ROBO1, OAS3, and STAT1 proteins that form a large complex in response to IFNα treatment (Fig. 4, Table S2)^32–34^. Most importantly, these interactions were detected in all three IFNα treated replicates, and were undetectable in untreated samples suggesting they form specifically in response to IFNα treatment. STAT1 is known to transcriptionally regulate expression of these ISGs, however, its interaction with ISGs at the protein level hasn’t been studied. The crystal structure of STAT1 reveals that its coiled-coil domain (CCD) is not involved in interactions with DNA or with the protomer when it forms dimer^35^. These α-helices form a coiled-coil structure that provides a predominantly hydrophilic surface area for interactions to take place^35^. In our CLMS data, we observed that most of the interactions with STAT1 are either in the CCD, linker domain or the SH2 domain prior to the C-terminal tail segment (residues 700–708) (Fig. 4A). A previous study reported that USP18 bound to the CCD and DNA binding domains (DBD) of STAT2 and was recruited to the type I IFN receptor subunit IFNAR2 to mediate suppression of type-I IFN signaling^24^. Our data also indicate that the catalytic domain of USP18 interacts with DBD of STAT1 (Fig. 4A,D) suggesting that STAT1 and STAT2 both may have a role in recruiting USP18 to IFNAR2.

There are two USP18 isoforms described in humans, full-length protein, which is mainly located in the nucleus, and an isoform lacking the N-terminal domain, USP18-sf that is evenly distributed in the cytoplasm and nucleus^36^. In addition, the N-terminus has been predicted to be unstructured and is not required for isopeptidase activity or ISG15 binding^37^. Most of the interactions identified in our study are situated in the N-terminus of the protein which suggests that these interactions involve full-length USP18 (Fig. 4A,D), and therefore, have a high probability of occurring in the nucleus. Moreover, our data also implies that the N-terminus is used exclusively for protein–protein interactions. The IFNAR2 binding site is located between residues 312–368 and it is interesting to note that none of the proteins in the complex bind to this region (Fig. 4A)^37,38^. Together the data suggest that the IFNAR2 binding region is used exclusively by the receptor protein. Additionally, only OAS3 and ROBO1 were found to be associated with both the N-terminus and the domain before the IFNAR2 binding site (Fig. 4A).

ROBO1 belongs to the immunoglobulin (Ig) superfamily of transmembrane signalling molecules and consists of five Ig and three fibronectin (Fn) domains in the extracellular region. These extracellular domains are followed by a membrane proximal region and a single transmembrane helix^39^. An unstructured intracellular region lies at the C-terminus, containing conserved sequence motifs that mediate the binding of effector proteins^39^. The region stretching from amino acids ~ 1100 to 1600 is mostly disordered. We found that MX1 interacted through ROBO1 via Ig, Fn, and intracellular domains while most of the interactions with STAT1 were between its CCD, linker domain, and the C-terminal of ROBO1 (Fig. 4A,E). On the other hand, interaction with DI, DIII, and the linker region of OAS3 was dispersed throughout the ROBO1 protein (Fig. 4A).

The oligoadenylate synthetase (OAS) family of proteins sense and bind to intracellular double-stranded RNA (dsRNA), undergo conformational change, and synthesize 2ʹ,5ʹ-linked oligoadenylates (2–5 As)^40^. Out of the three OASs, OAS3 has been found to display higher affinity for dsRNA and to synthesize minimal 2–5 As which can activate RNase L, and thereby, restrict viral replication^41^. The OAS family consists of polymerase beta (pol-β)-like nucleotidyl transferase domains. A previous study showed that the catalytic activity of the C-terminal domain (DIII) is dependent on the dsRNA-binding domain (DI) that is essential for activation of OAS3^42^. We observed that the DI and DII domain of OAS3 interacted with the CCD and a small linker region between SH2 and TAD of STAT1 (Fig. 4A,F). Overlay of different cross-linked sites over the protein structures shows interaction between the β-sheets and loops of the STAT1 DBD with the exposed pocket or cavity formed by residues 60–75 in the DI domain of OAS3 (Fig. 4G). Orientation of the protein in the complex also showed that none of the interactions with OAS3 interfered with the DNA binding ability of its DI domain (Fig. S1A). In addition, the N-terminal GTPase domain of MX1 interacts extensively with both DI and DIII domains of OAS3 (Fig. 4A). We also observed an interaction between OAS1 and MX1 in all three IFNα treated replicates, where the only domain of OAS1, which is also catalytically active, interacts with all the three domains of MX1 (Fig. S2A,B).

MX proteins are part of large dynamin-like GTPase family that contains a N-terminal GTPase domain that binds and hydrolyses GTP, a self-assembly-mediating middle domain, and a C-terminal leucine zipper (LZ) domain which acts as a GTPase effector domain^25,43^. MX1 associates with subunits of the viral polymerase to block viral gene transcription^43^. A previously reported yeast two-hybrid screen revealed that MX1 bound to PIAS1 inhibits STAT1-mediated gene activation by blocking the DNA binding activity, and also has SUMO E3-ligase activity^44,45^. Here we demonstrate that MX1 binds to STAT1 (Fig. 4C,D), however, how this interaction affects STAT1-mediated gene activation in response to IFNα needs further investigation. Additionally, we also found MX1 interacting with IFIT3 and DDX60 in all three IFNα treated replicates (Fig. S2C).

DDX60, an IFN-inducible cytoplasmic helicase, has been previously reported playing a role in RIG-I-independent viral RNA degradation^46^. It interacts with RIG-I and activates its signalling in a ligand-specific manner^46^. DDX60 consists of a DEXD/H-Box helicase domain which binds to viral RNA and DNA, and a C-terminal helicase domain^47^. Most of its interactions with MX1 and IFIT3 were within the long N- and C-terminal regions with no typical domains or motifs (Fig. S2E,F). However, MX1 was also linked with the DEXD/H-Box helicase domain (Fig. S2E). IFIT family proteins have distinguished tandem copies of helix-turn-helix motifs called tetratrico-peptide repeats (TPRs). IFIT3 was found to be a positive modulator of RIG-I signaling, and hence, a component of MAVS complex^48^. Together, our data suggests that IFIT3 and DDX60 interact with each other mainly in the region between TPR 3–6 of IFIT3, and may have a role in RIG-I/MAVS signalling (Fig. S2F).

### Antigen presenting MHC-I molecule and its interaction network

**Figure 5:**
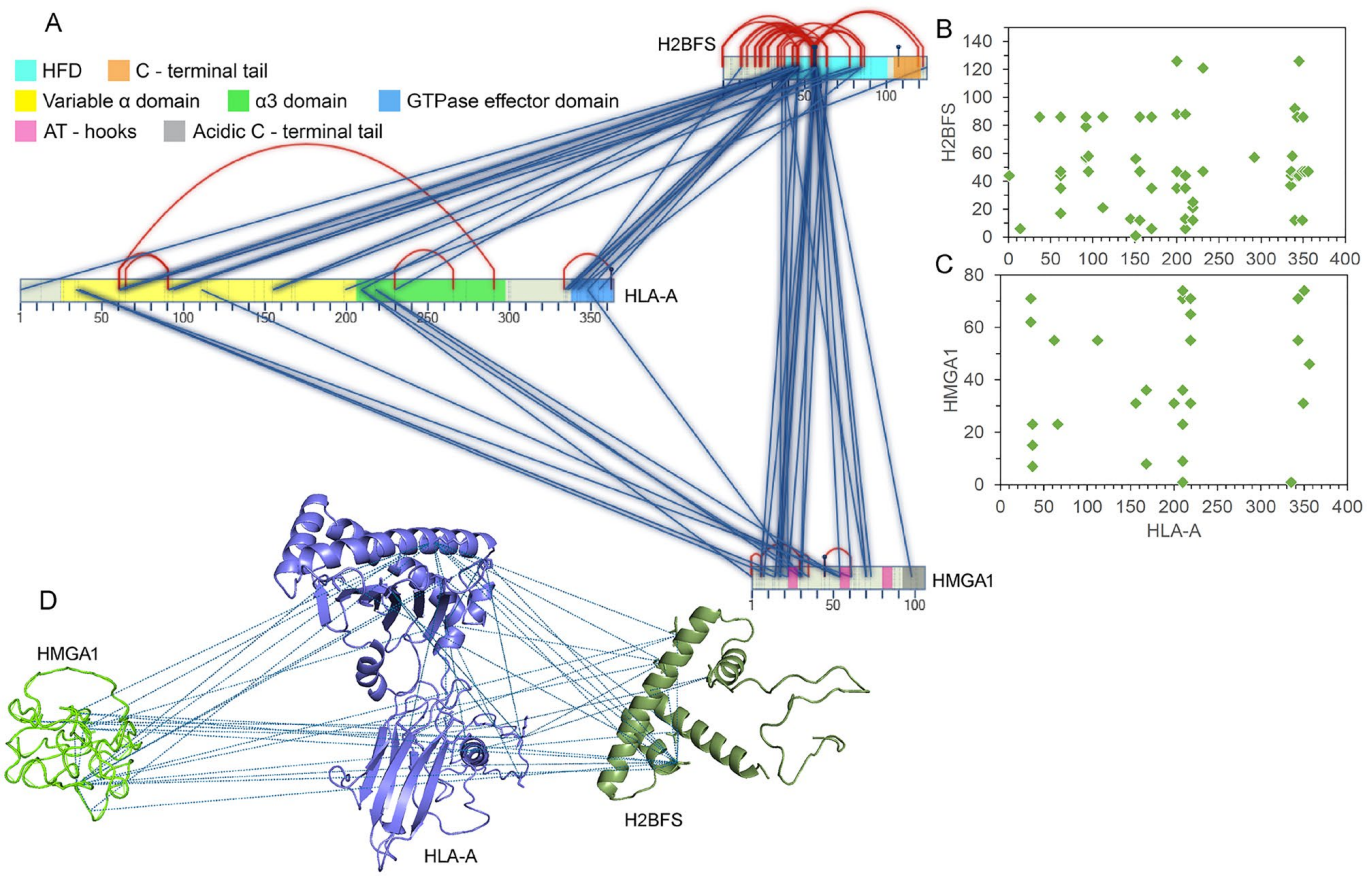
The IFNα induced interaction network of HLA-A with H2B (H2BFS) and HMGA1. **(A)** 2D map (generated in SIM-XL program^29^) depicting different types of interaction within the H2B-HLA-A-HMGA1 complex; inter-link (blue), intra-link (red) and mono-link (black). Domains of different identities are labeled with their respective colors^32^: H2B (histone; 2–102) and MHC-I (MHC_1; 25–203, C1-set; 210–290, and MHC_I_C; 337–364). The cross-link score cut-off was set to 3.5. Dot plots representing interaction sites of HLA-A with H2B **(B)** and HMGA1 **(C)**, with interaction site K or S between two peptides. In the plots, the cross-link score cut-off was set to 3.0. **(D)** Inter-links between proteins displayed over the H2B, HLA-A, and HMGA1 protein structures in the PyMOL program. These structures were modeled using the Phyre2 server (http://www.sbg.bio.ic.ac.uk/phyre2), and the template structures used for the proteins H2B, HLA-A, and HMGA1 were 1kx5^52^, 1kj3^49^, and 2eze^55^, respectively.

**Figure 6:**
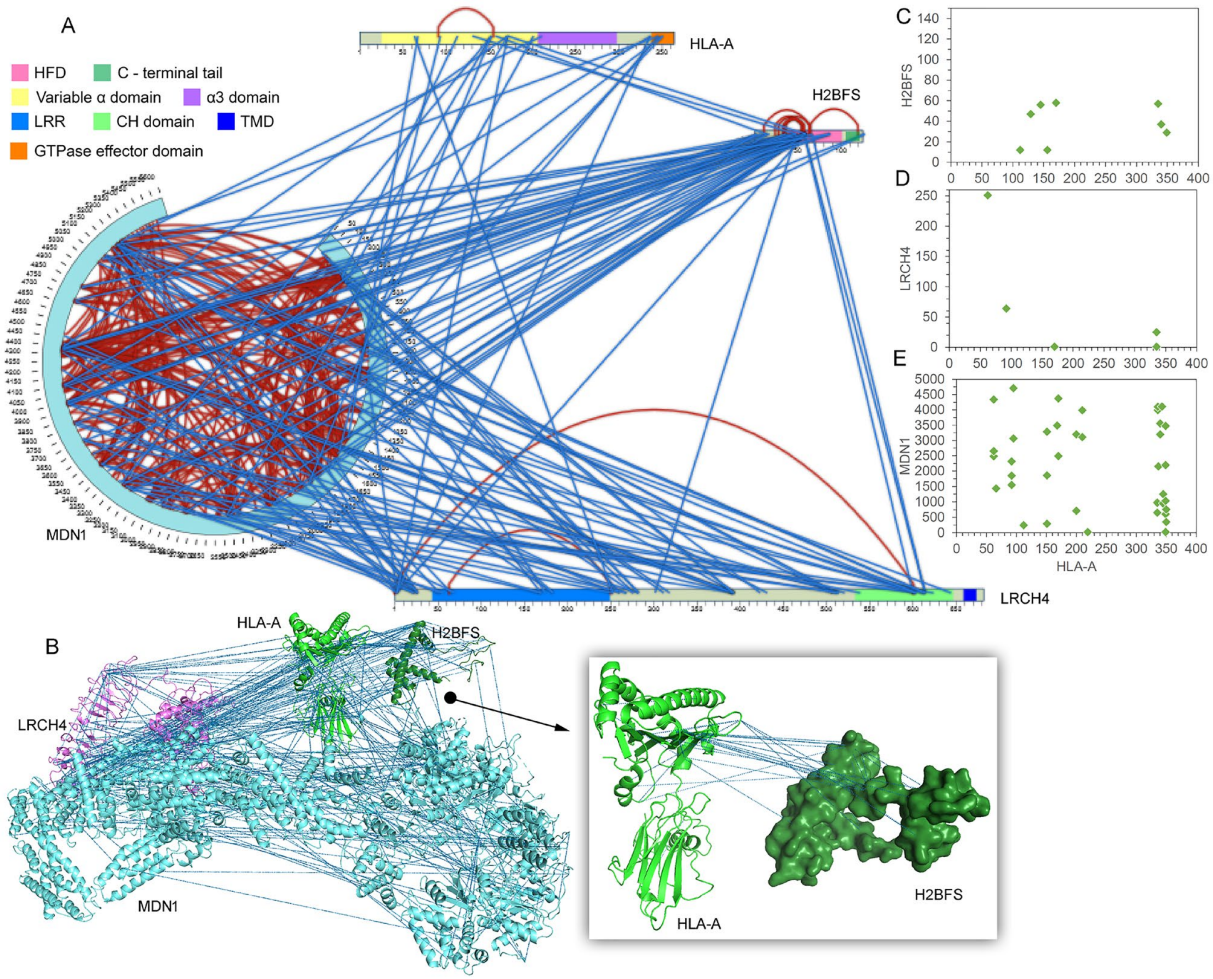
The IFNα induced interaction network of HLA-A with H2B (H2BFS), MDN1, and LRCH4. **(A)** The intramolecular (red) and intermolecular (blue) cross-links represented in the 2D interactive map (generated in SIM-XL program^29^), MDN1 is presented in the circular view. The cross-link score cut-off was set to 3.5. Domains of different identities are labeled with their respective colors^32^: H2B (histone; 2–102), MHC-I (MHC_1; 25–203, C1-set; 210–290, and MHC_I_C; 337–364), and LRCH4 (LRR_8 (68–126), LRR_8 (137–194), and CH (535–641)). **(B)** Inter-link between proteins displayed over the H2B, HLA-A, LRCH4, and MDN1 protein structures in the PyMOL program. These structures were modeled using the Phyre2 server (http://www.sbg.bio.ic.ac.uk/phyre2), and the template structures for the proteins H2B, HLA-A, LRCH4, and MDN1 were 1kx5^52^, 1kj3^49^, 6hlu^62^, and 6i26^65^, respectively. Dot plots showing the interaction sites K or S of HLA-A with H2B **(C)**, LRCH4 **(D)**, and MDN1 **(E)**. For the plots, the cross-link score cut-off was set to 3.0.

Considering proteome-wide screening is computationally demanding, we next screened the entire human UniProt database for one of the IFNα treated replicates. We found some high-confidence interaction networks for HLA-A in that replicate. The pathway analysis for identified proteins from MS/MS spectra revealed MHC-I based antigen processing and presentation as the dominant pathway induced by interferon (Fig. 3D). Therefore, we focused on exploring high-confidence protein interactions of MHC-I molecules across all the cross-linked samples. HLA consists of α1, α2, and α3 domains and a light chain, β2 microglobulin (β2m) being a constant protein partner^49^. HLA is unstable in the absence of peptide ligand following its assembly in the endoplasmic reticulum^50^. The peptide binding groove is formed by α1 and α2 domains that are highly polymorphic and unstructured in the peptide-free form, and a α3 domain that is comparatively less polymorphic^51^. In the presence of IFNα, we found two HLA-A complexes: one that interacts with HMGA1 and H2B (Fig. 5, Table S3) and another where it interacts with MDN1, LRCH4 along with H2B (Fig. 6).

Apart from maintaining genome integrity, histone H2B is involved in the transcriptional regulation. H2B protein is composed of a central histone-fold domain (HFD) formed by three alpha helices separated by loops, and a C-terminal tails^41,52^. The majority of interactions with H2B were in the α1-helix that mediates trimerization with the HFD heterodimer (Fig. 5A,B). Even though lysines are involved in DNA-binding, some of the lysines are also sites for alternative acetylation or methylation. For instance, the residues K43, K46 and K57 from the H2B are not involved in direct DNA-binding but are targets of different post-transcriptional modifications^53^. Similarly, K44, K47 and K57 residues in H2B may have an alternative role in presence of IFNα, which includes interacting with other proteins (Fig. 5A,B). Moreover, extrachromosomal histone H2B activates immune responses in various cell types acting as a cytosolic sensor to detect double-stranded DNA (dsDNA) fragments derived from infectious agents or damaged cells^54^. Depletion of H2B suppressed IFN-β production and STAT1 phosphorylation in presence of DNA viruses^54^. H2B is also known to travel in and out of the nucleus more rapidly than other core histones^54^. H2B interaction with MDN1 and with LRCH4 were also observed in individual untreated samples. We found HLA-A interacting with H2B in all three IFNα treated samples and one untreated replicate. This data reflects the role of H2B in alternative physiological functions, independent of transcriptional regulation.

HMGA1 (High Mobility Group AT-Hook 1), a small nuclear protein enriched in disorder-promoting amino acids, was identified in complex with HLA-A. It has an acidic C-terminal tail and three differentially spaced DBDs, called AT-hooks as they bind to minor grooves of AT-rich regions in dsDNA^55,56^. This binding induces bending or straightening of DNA allowing the access of canonical transcription factors to their consensus sequences. The C-terminal tail is assumed to be involved in protein–protein interaction and recruitment of transcription factors since a C-terminal deletion mutant fails to initiate transcription^57^. Moreover, this domain contains several conserved phosphorylation sites that are known kinase substrates^58^. We observed the interactions of HLA-A and H2B with HMGA1 outside the C-terminal domain, suggesting the C-terminal domain is mainly used for transcription factor binding (Fig. 5A,C). HMGA proteins compete with histone H1 for binding to linker DNA, thereby resulting in increased accessibility^57^. Similarly, it is plausible that HMGA interacts with histone H2B along linker DNA while competing with histone H1. HMGB1 induces HLA-A, -B, and -C expression in dendritic cells leading to their activation^59^, nevertheless, an interaction between HMGs and HLAs hasn’t been reported previously. We found that HMGA1 interacts with the α1 and α3 domains of HLA-A with most of the interaction outside its 3 DBDs (Fig. 5A,C). In our hands, HLA-A was found to be localized in the nucleus (data not shown), and given that H2B and HMGA1 also reside in the nucleus, there is a high probability that this interaction occurs in the nucleus. The specific adducts measured between H2B, HLA-A, and HMGA1 are described in Fig. 5D.

The majority of HLA-A interactions with other proteins are localized at its α1 and α2 domains as well as within the disordered C-terminal domain (Fig. 6). In one of these examples, we found that HLA-A interacts with the disordered N-terminal tail of LRCH4 (Fig. 6A,D). LRCH4 regulates TLR4 activation and cytokine induction by LPS, and therefore, regulates the innate immune responses^60,61^. It is a membrane protein with nine Leucine-rich repeats (LRRs) and calponin homology (CH) motif in its ectodomain, followed by a transmembrane domain (TMD)^60,62^. The CH domain is reported to mediate protein–protein interactions^60^. A stretch of around 300 amino acids between the LRR and CH domain is relatively accessible but disordered. In line with the function of disordered regions as mediators of protein–protein networks and vesicle trafficking^63^, we found most of the protein interactions in the disordered region. Interaction with MDN1 was dispersed throughout the protein's length including LRR1, LRR6, CH domain, and disordered region while H2B was bound mostly with CH domain (Fig. 6A,B). It is noteworthy that none of the interactions involved the TMD which shows the specificity of the CLMS method (Fig. 6A,B).

MDN1 was also identified as a part of the HLA-A protein network (Fig. 6A). It belongs to the AAA protein family (ATPase associated with various activities). It’s identical N-terminal AAA domains orchestrate into hexameric rings and remove assembly factors from ribosomal 60S subunit^64^. Cryo-EM studies in yeast reveal that AAA domains are followed by six non-equivalent AAA domains linked in a single polypeptide which appears similar to dynein^64–66^. Further, a stretch of Asp/Glu-rich region is followed by a MIDAS (metal ion-dependent adhesion site) domain. Owing to MDN1’s large size (~ 5600 amino acids) and its limited homology to well-studied proteins, not a lot is known about its structure and function in humans. We identified HLA-A, H2B, and LRCH4 as binding partners of MDN1 and their orientation as a protein complex was revealed in PyMol (Fig. 6A,B). These three proteins interact with AAA domains, dynein-like linker domain and probable MIDAS domain of MDN1. In a previous report, affinity purification of the bait proteins identified MDN1 as the protein associated with histone H2B^67^. Moreover, a recent study has also reported interaction between MDN and HLA-B using affinity-purification mass spectrometry in HCT116 cells which supports our findings^68^. Identification of this complex in IFNα treated samples implies that MDN1 has a role in interferon signalling.

As HLA genes are highly polymorphic, we extracted sequencing reads mapping to HLA-A, -B and -C from the RNA-seq data of the Flo-1 cells (data not shown). Peptide sequences corresponding to sequencing reads showed a significant difference among HLA-A, -B and -C in the regions where the cross-linked peptides reside in HLA-A (Fig. S3). Moreover, we did not observe protein–protein cross-links for the HLA-B/C molecules with either of the H2B/HMGA1/MDN1/LRCH4 proteins. This suggests that the protein interaction found between HLA-A, MDN1, LRCH1, and HMGA1 is specific to HLA-A. Additionally, the proteomics analysis of non-cross-linked samples (Table S4) suggests that HLA-A is enriched with higher sequence coverage compared to that of the HLA-B or HLA-C. The peptides identified for HLA-A have high intensities in both IFNα treated and untreated samples.

### Validation of novel HLA-A binding proteins

**Figure 7:**
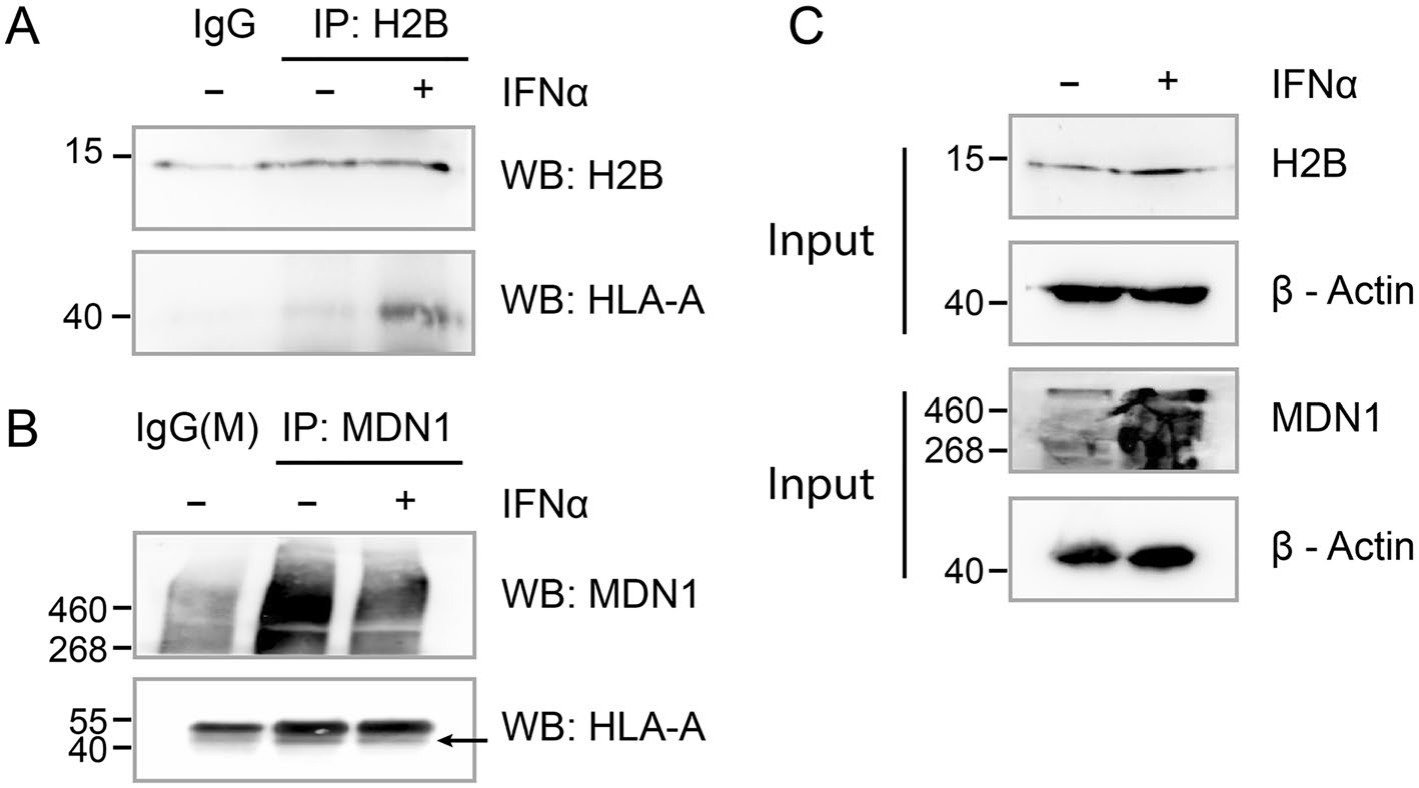
HLA-A co-purifies with H2B and MDN1. Representative immunoblotting of endogenous H2B **(A)** and MDN1 **(B)** immunoprecipitated from IFNα treated Flo-1 cells and probed with the indicated antibodies. Mouse and rabbit IgGs were used as negative control. **(C)** The relative amount (Input) of the different antigens were depicted by immunoblots probed against indicated antibodies and β-actin was used as a loading control.

To ensure that the interactions identified here are not due to non-specific cross-linking of two proteins in close spatial proximity, we further validated two novel interactors of HLA-A by performing co-immunoprecipitation assay. Interactions of HLA-A with endogenous MDN1 and H2B were detected in IFNα treated and untreated Flo-1 cells (Fig. 7, Fig. S4). We confirmed that HLA-A was captured with H2B in immunoprecipitates, and this association was induced by IFNα treatment as HLA-A was absent in immunoprecipitated samples from untreated cells (Fig. 7A). However, our data demonstrate that IFNα differentially regulates HLA-A binding to H2B and MDN1. IFNα induced association between H2B and HLA-A but decreased its binding with MDN1. We found that MDN1 associates with HLA-A in control samples, while the addition of IFNα reduces this interaction irrespective of MDN1 induction by IFNα (Fig. 7B,C). Moreover, immunoprecipitation of HLA-A captured H2B in A549 cells (Fig. S4) suggesting this interaction is not cell type-dependent. Together, these results confirm interferon-mediated interaction of HLA-A with H2B and MDN1.

### The conformational dynamics of the HLA-A binding proteins

**Figure 8:**
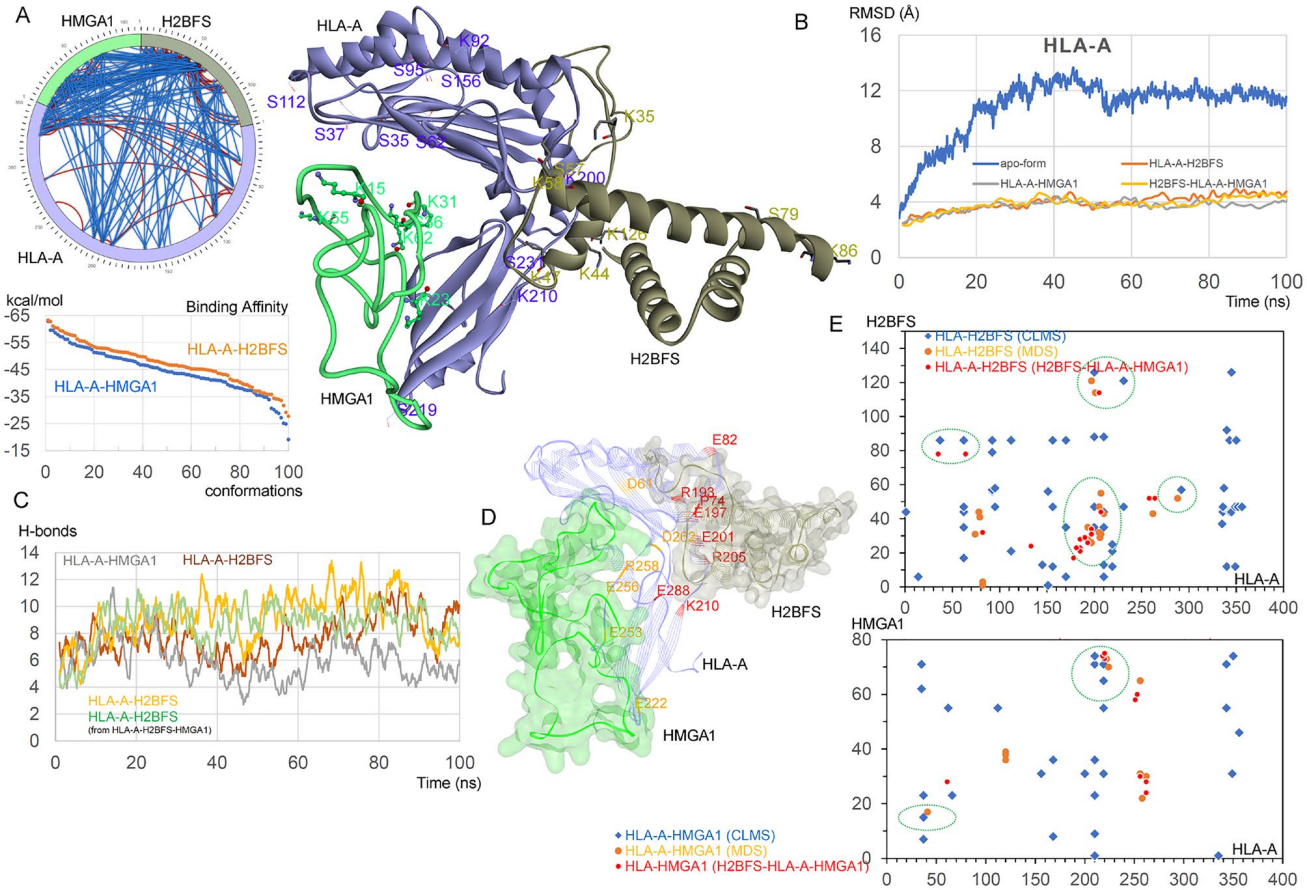
Conformational dynamics of the possible network between H2B (H2BFS)-HLA-A, HMGA1-HLA-A and H2B-HLA-A-HMGA1 complexes. **(A)** The left panel represents a 2D map (generated in SIM-XL program^29^) of intramolecular (red) and intermolecular (blue) cross-links (cross-link score cut-off was set to 3.5). In addition, the identified cross-link residues were labeled on the H2B, HLA-A, and HMGA1 protein structures. The binding conformations of these proteins were retrieved by the docking pipeline implemented in the MOE package. The bottom left panel shows different possible conformations of the H2B-HLA-A and HMGA1-HLA-A complexes, varying in their protein–protein binding affinity (GBVI/WSA dG; kcal/mol). **(B)** The root-mean-square deviation (RMSDs) of atomic positions (excluding hydrogen atoms) of each protein structure. **(C)** Intermolecular protein–protein hydrogen-bond interactions from different simulated complexes, a particular interaction lasting ≥ 10 ns were considered. The h-bond donor–acceptor atom cutoff distance was set to 3.5 Å, and the donor-H-acceptor angle cutoff was set to ≥ 160°–180°. **(D)** Labeled residues forming protein–protein interactions from HLA-A with respective partners with occupancy ≥ 20 ns, retrieved from the HLA-A-H2B and HLA-A-HMGA1 simulated complex. The protein structure represents averaged structures from 100 ns MDS. **(E)** Differently identified cross-links between the HLA-A-H2B and HLA-A-HMGA1 complexes based on the interaction site K or S between two peptides, compared with the interactions traced from 100 ns MS simulations of the H2B-HLA-A/HMGA1-HLA-A/H2B-HLA-A-HMGA1 complexes. The cross-link score cut-off was set to 3.0, and particular interactions from MDS having occupancy ≥ 10 ns were considered. Protein structures were visualized using the BIOVIA Discovery Studio (Dassault Systèmes, BIOVIA Corp., San Diego, CA, USA) and Molecular Operating Environment (MOE; Chemical Computing Group Inc., Montreal, QC, Canada) packages.

Structural properties of one of the high-confidence cross-linked networks induced by interferon—H2B-HLA-A-HMGA1 was investigated. We took advantage of molecular dynamics simulation as an alternative approach to understand the conformational dynamics of the proteins involved in this complex (Fig. 8). Findings from CLMS data suggest the probability of different conformations between H2B, HLA-A, and HMGA1 proteins. Therefore, the following potential complexes were simulated in the solvent environment: H2B-HLA-A, HMGA1-HLA-A, and H2B-HLA-A-HMGA1. Initial protein–protein docking screening using the MOE package (Molecular Operating Environment; Chemical Computing Group Inc., Montreal, QC, Canada) proposed different possible conformations between these proteins (Fig. 8A). Visualization of docked protein complexes revealed several interactions and possible conformations (Figs. 5A, 8). As such, one of the possible conformations is represented in Fig. 8A (with labeled cross-links), which was further evaluated with the MD simulation pipeline. In addition, the binding energies of H2B or HMGA1 with HLA-A highlight that H2B has higher affinity with HLA-A (Fig. 8A).

Stability of the HLA-A molecule over time (root-mean-square deviation; RMSDs or root-mean-square fluctuations; RMSFs) suggests that the presence of H2B or HMGA1 protein in the complex stabilizes HLA-A (Fig. 8B, Fig. S5). The HMGA1 protein binding closely to the B2M site of HLA-A, induces stability in the HLA-A amino acids in both the complexes the HLA-A-HMGA1 or H2B-HLA-A-HMGA1 (Fig. 8B, Fig. S5). Particularly, HLA-A residues ~ 60–90 and ~ 180–210 were found exhibiting lesser flexibility in the presence of H2B (Fig. 8B). Both H2B and HMGA1 display better binding with the HLA-A in the H2B-HLA-A-HMGA1 complex, compared to HLA-A binding to H2B or HMGA1 alone (Fig. 8C,D; Table S5). Residues involved in hydrogen bond formation (MD simulation high occupancy ≥ 10 ns) coincide with CLMS cross-link (K or S residues) interaction sites in the complex which suggests high confidence in interactions identified through the CLMS method (Fig. 8E). HLA-A residues between ~ 190–210 and ~ 200–220 amino acids were found binding H2B and HMGA1, respectively, in the CLMS and MD simulation (Fig. 8E).

## Discussion

The protein–protein interactions form into dynamic structural networks that mediate intracellular communication in response to some stimulus. Because many proteomics approaches define changes in total steady-state protein levels, protein–protein interaction dynamics need additional tools to capture binding interfaces, CLMS is one such tool. The interferon signaling system constitutes a cytokine network that allows cells to respond to a range of environmental pathogenic and internal pathological signals, eventually leading to induction of a subset of interferon-induced proteins. We applied CLMS to determine whether novel protein–protein interactions can be defined within the set of interferon induced proteins. Global protein cross-linking analysis in the interferon responsive Flo-1 cell model was used to trap protein complexes. Tryptic peptides were recovered from non-cross-linked and cross-linked cells allowing for peptide counts, pathway enrichment, and distribution of peptide length plus defined LFQ intensities. Classic interferon induced proteins were identified as a positive internal control, whilst novel inter- and intra- molecular cross-linked adducts were observed for canonical interferon induced proteins, such as MX1, UP18, OAS3, and STAT1. Different structural features and interactions residing in functional regions were investigated.

Interactions between HLA-A, MDN1, and H2B were detected with immunoblotting in IFNα treated and untreated Flo-1 and A549 cells. Our findings highlight that HLA-A forms a complex with H2B in an IFNα-dependent manner. Our work presents an interesting avenue to further explore colocalisation of these two complexes. It will also be interesting to deploy the CLMS approach on a panel of cell lines to identify cell type-independent interferon-mediated protein interactions. We finally took advantage of MD simulations as an alternative approach to understanding the conformational dynamics of the proteins involved in the H2BFS-HLA-A-HMGA1 complex where both intra- and inter-molecular cross-link interactions were traced. Findings from CLMS data suggest the probability of different conformations between H2BFS, HLA-A, and HMGA1 proteins. Different possible conformations between these docked protein complexes revealed several interactions similar to those observed in the CLMS datasets. One of the main advantages of our approach is that it can easily identify interactors of highly polymorphic genes such as HLAs, therefore, it would be interesting to explore HLA-haplotype specific protein interactions which are otherwise difficult to study. Together, our data show that CLMS can be used to expand our knowledge on the interferon induced signalling networks and form a foundation for studying more complex cell–cell systems within the tumour microenvironment.

## Methods

### Cell culture and cross-linking reaction using DSS

Flo-1 cells were obtained from ATCC and maintained in DMEM (Gibco) supplemented with 1% penicillin/streptomycin (Invitrogen), 10% fetal bovine serum (Gibco) and incubated at 37 °C with 5% CO2. Cells were grown to 70–80% confluence prior to treatment with IFNα14 (produced by Edinburgh Protein Production Facility). All other chemicals and reagents were obtained from Sigma Aldrich unless indicated otherwise.

The Flo-1 cells were cultured in a 6-well plate and the next day cells were treated with 10 ng/ml IFNα14 for 24 h until ~ 80% confluency. The cells were washed thrice with PBS and were cross-linked for 5 min at 37 °C using freshly prepared DSS (Thermo Fisher Scientific) (dissolved in DMSO) in PBS to achieve 0.5 mM final concentration. The DSS cross-linking reaction was replaced with PBS and the residual DSS was quenched by addition of 20 mM Tris (pH 8.0) in PBS for 15 min at 37 °C. The cells were harvested by scrapping and collected in a low-binding tube (Axygen).

### Two steps cross-linked protein solubilization

Cell pellets were lysed with 300 µl urea lysis buffer (8 M Urea, 0.1 M Tris, pH 8.5) at room temperature for 30 min with occasional vortex. All the centrifugation steps were performed at 14,000×*g* at 8 °C. The lysates were centrifuged for 10 min, and the supernatant was transferred into a new tube. The remaining transparent pellet was solubilized with 150 µl second lysis buffer (2 M Urea, 2% (w/v) SDS (sodium dodecyl sulphate)) for 30 min or longer until uniformly aqueous solution was obtained. The lysates were centrifuged for 20 min, and the supernatant was pooled with lysate retrieved in the previous step. Protein concentration was assessed using the Micro BCA assay (Thermo Fisher Scientific) according to the manufacturer’s instructions for the microplate procedure. Samples were snap frozen in liquid nitrogen and stored at − 80 °C.

### Peptide generation using FASP

Approximately 100 μg of soluble cross-linked protein was processed using a modified filter-aided sample preparation (FASP) protocol as described in Wisniewski et al*.*^69^ Briefly, cross-linked proteins were mixed with 200 µl of Urea buffer (8 M Urea in 0.1 M Tris pH 8.5), vortexed, and split into two halves. All the centrifugation steps were performed at 14,000×*g* at 25 °C. The first half of cross-linked protein lysate was transferred to a 10 kDa cut-off Microcon Centrifugal Filter Unit with Ultracel-10 membrane (Merck) followed by filter centrifugation for 25 min. Next, the second half of protein was added to the filter, and the same procedure was repeated. Protein reduction was performed by adding 100 µl of 17 mM Tris (2-carboxyethyl) phosphine hydrochloride (TCEP) in the urea buffer. The reduction reaction was held on a thermomixer at 37 °C for 30 min with agitation at 600 rpm. Further, the column was centrifuged and the reduced cross-linked proteins were alkylated using 100 µl of 50 mM iodoacetamide in the urea buffer. Alkylation reaction was carried out in the dark for 20 min at RT. The column was centrifuged, and the column walls were rinsed three times with 100 µl of urea buffer, followed by centrifugation. The same procedure was performed three times with 100 µl of 100 mM ammonium bicarbonate. The collection tube was replaced with a new one prior to tryptic digestion. Digestion buffer comprising 50 mM ammonium bicarbonate was added along with 1 µl of trypsin diluted in the trypsin buffer (Promega). Trypsin to protein ratio was kept at approximately 1:33, and the digestion reaction was incubated overnight at 37 °C in a humidified chamber. Cross-linked peptides were eluted from the filter by centrifugation for 25 min. Peptide recovery was enhanced by adding 50 µl of 0.5 M NaCl to the filter, followed by centrifugation for 25 min.

### Peptide desalting

C18 Micro Spin Columns (Harvard Apparatus) were used to desalt cross-linked tryptic peptides using a protocol described in Bouchal et al*.*^70^ with minor changes. Briefly, C18 spin columns were activated by three washes of 0.1% formic acid (FA) in acetonitrile (AcN) (Merck) and two washes of 0.1% FA. The column was hydrated for 15 min by 0.1% FA. The sample was loaded into the spin column and washed three times with 0.1% FA. Desalted peptides were eluted by a step gradient using 50%, 80%, and 100% AcN in 0.1% FA consecutively. Samples were dried in SpeedVac Concentrator plus (Eppendorf) until no residual liquid was present. Eluted peptides were dissolved in 100 µl of 0.08% trifluoroacetic acid in 2.5% AcN and concentration was measured on NanoDrop 2000 (Thermo Scientific). Approximately 1 µg of cross-linked peptides from each sample was injected into the LC–MS/MS system.

### Peptide separation and data-dependent mass spectrometry acquisition

Cross-linked peptides were separated on UltiMate 3000 RSLCnano LC System (Thermo Scientific) connected to Orbitrap Exploris 480 Mass Spectrometer (Thermo Scientific). Cross-linked peptides were concentrated on μ-precolumn C18 trap cartridges 300 μm inner diameter (ID) and 5 mm length packed with C18 PepMap100 sorbent with PepMap 5 μm sorbent (Thermo Scientific). Loading pump flow was set to 5 μl/min of 0.08% trifluoroacetic acid in 2.5% AcN. Cross-linked peptides were separated on 75 μm ID and 150 mm long fused-silica analytical column packed with PepMap 2 μm sorbent (Thermo Scientific). The mobile phase A and B were composed of 0.1% FA in water and 0.1% FA in AcN respectively. The gradient started at 2.5% B linearly increasing up to 40% B in 90 min, followed by a linear increase up to 90% B in the next 2 min. Mobile phase composition remained at 90% B for 10 min, followed by a linear decrease to 2.5% B in 2 min. Column equilibration before the next run was performed at 2.5% B for 8 min. Cross-linked peptides eluted from the analytical column were ionized in a nano-electrospray ion source (NSI) and were introduced into Exploris 480 mass spectrometer (Thermo Scientific).

The Orbitrap Exploris 480 mass spectrometer was operated in positive data-dependent mode. Full-scan was operated in profile mode with 120,000 resolution, and the range was set from m/z 350 Th to m/z 2000 Th. The normalized AGC target was set to 300% with 50 ms maximum injection time. Monoisotopic peak detection was set to Peptide. A setting to relax restrictions if too few precursors are found was set to true. Minimum precursor ion intensity was set to 5.0e3 and precursor charge states up to + 8 were included in the experiment.

Cycle time data-dependent mode was set to 2.5 s between master scans. The dynamic mass exclusion was set to 20 s after the first precursor ion fragmentation. The precursor isolation window was set to 2 Th. Normalized collision energy type with fixed collision energy mode was selected in data-dependent MS/MS scan. The collision energy was set to 30%. Orbitrap resolution was set to 15,000, and the AGC target was set to 100%. Custom maximum injection time setting was set to 60 ms.

### Database search and analysis

Prior to tracing protein–protein networks in the cross-linked samples, we processed the raw files to identify peptides/proteins traced in the samples using the MaxQuant package (version 1.6.12.0)^26,27^. In addition, similar proteomics analysis was performed for non-cross-linked IFNα treated and untreated Flo-1 samples. The MS/MS data was searched against human UniProt (www.uniprot.org) database (downloaded on 12.08.2020 consisting of 75,093 entries) using the built-in andromeda search engine^27^. The search was performed without specifying enzyme specificity and variable modifications for deamidation (N, Q) and oxidation (M). The precursor mass tolerance was set to 20 ppm and product ions were set at 0.02 Da. The initial and the maximum mass deviation was set to 10 ppm. Maximum mass of a peptide was set to 4600 Da and sequence similarity was set between 7 and 25 amino acids (aa). Further statistical analysis was performed using the Perseus program (version 1.6.10.45). The protein abundances were computed by normalized spectral protein intensity (LFQ intensity; label-free quantification)^27^, and the intensity values were Log2 transformed. Hierarchical clustering of the proteins identified against their peptide intensities were mapped using pheatmap (v1.0.12) package in R (v 4.1.2). Pathway enrichment analysis using the Reactome pathway database was carried out for the proteins that were upregulated more than fourfold with IFNα treatment compared to untreated samples.

### Identification of cross-links and protein structure modeling

Identification of Lysine (K) or Serine (S) specific chemical cross-linking of protein complexes traced using LC–MS/MS were performed using the Spectrum Identification Machine for Cross-Linked Peptides (SIM-XL)^29^. First, possible interactions were searched between the Interferon (IFN)-related DNA damage resistant signature (IRDS) genes using a dataset of IRDS proteins described in Padariya et al.^28^. Screening the whole human UniProt for all the conditions and replicates is computationally very demanding, therefore, the entire human UniProt (www.uniprot.org) database (downloaded on 12.08.2020 consisting of 75,093 entries) was screened for one of the IFNα treated replicate to obtain high-confidence interactions. These high-confidence interactions obtained were taken forward and screened in all the replicates and conditions.

In SIM-XL, the cross-linker (XL) was set to DSS, the XL mass shift and modification mass shift was set to 138.06 and 156.07, respectively. The following cross-link reaction sites were considered: KK, KS, and KN-TERM, with no reporter ions. Both precursor and fragment ppms were set to 20, and the Xrea threshold to 0.15. Trypsin enzyme was considered fully specific, and the Higher-energy C-trap dissociation (HCD) fragmentation method was implemented. The dynamic DB reduction XCorr threshold and the dynamic DB reduction minimum number of peptides were set to 2.5 and 2, respectively. Other parameters were the following: single isotopic possibilities and peaks matched cutoff, 4 minimum AA residues per chain and intra-link maximum charge, and 3 maximum missed cleavages. Obtained cross-linked 2D maps were analyzed in (SIM-XL), as well as xQuest^28^ graphical representations were considered to plot 2D maps. The protein–protein cross-links over the protein structure were represented in PyMol (The PyMOL Molecular Graphics System, Version 2.0 Schrödinger, LLC).

Using the homology modeling principles and implementing the “Hidden Markov method” the protein model structures were generated using the Phyre2 server (http://www.sbg.bio.ic.ac.uk/phyre2)^11^. Phyre2 generates model structures considering the sequence alignments with the known protein structures. For the proteins H2BFS, HLA-A, HMGA1, LRCH4, and MDN1 the template structures were 1kx5^52^, 1kj3^49^, 2eze^55^, 6hlu^62^, and 6i26^65^. In addition, AlphaFold^71^ structures for MX1, UBP18, and ROBO1 were considered. The BIOVIA Discovery Studio Visualizer (Dassault Systèmes, BIOVIA, San Diego, CA, USA) as well as the Molecular Operating Environment (MOE; Chemical Computing Group Inc., Montreal, QC, Canada) packages were used for protein structure visualization.

### Molecular dynamics simulations of the protein–protein network

Protein–protein docking screen was performed using the pipelines implemented in the Molecular Operating Environment (MOE; Chemical Computing Group Inc., Montreal, QC, Canada) package applying the CHARMM27 forcefield^30^. Individual screens between H2BFS-HLA-A and HMGA1-HLA-A were ranked based on the Generalized Born/Volume Integral (GBVI/WSA dG; kcal/mol) score^72^. Docking conformations corresponding to the CLMS binding sites were further investigated using the molecular dynamics simulation approach (MDS). Applying the CHARMM27 forcefield in the GROMACS 4.6.5^31^ package, the following 6 different systems were investigated: H2BFS (apo-form), HLA-A (apo-form), HMGA1 (apo-form), H2BFS-HLA-A, HMGA1-HLA-A, and H2BFS-HLA-A-HMGA1. MD simulation was performed using simple point charge (SPC) water molecules^31^ and, Na+ and Cl− counter ions were added in the periodic boundary conditions imposed in the simulation box. Initially, each system was minimized for 50,000 steps of the steepest descent algorithm (temperature 300 K; V-rescale thermostat^31^ and pressure 1 bas; Parrinello-Rahman barostat^31^). Further, the production run was performed for 100 ns (leapfrog integrator^31^). MDS data were visualized in VMD (Visual Molecular Dynamics) tool^73^ and BIOVIA Discovery Studio (Dassault Systèmes, BIOVIA Corp., San Diego, CA, USA).

### SDS-PAGE and Coomassie staining

The Flo-1 cells were washed twice with cold PBS and harvested by scraping. Cell pellets were lysed with urea lysis buffer (7 M urea, 25 mM HEPES pH 7.5, 25 mM NaCl, 5 mM DTT and 0.05% (V/V) Triton X-100, 1× protease inhibitor cocktail). Protein concentration of lysed samples was quantified using Protein Assay Dye Reagent (Bio-Rad), according to the manufacturer’s instructions. Proteins were resolved on 8% polyacrylamide gels and stained with Coomassie stain solution (50% water, 40% methanol, 10% acetic acid, 0.1% Coomassie brilliant blue; CBB) for 30 min. Later, the gel was destain overnight in the stain solution excluding CBB.

### Immunoblotting and co-immunoprecipitation

For immunoblotting, the samples were processed for SDS-PAGE and protein samples were resolved on either 8% polyacrylamide gels or on Biorad 4–15% precast gel (for Co-IP samples) and transferred onto 0.2 μM nitrocellulose membranes (Amersham Protran, GE Healthcare). Immunoblots were blocked with 5% skimmed milk powder in PBST (PBS + 0.1% Tween-20) and then probed with primary antibodies against MDN1, H2BFS, HLA-A (rabbit: Thermo Fisher, PA5-29911 and mouse: Origene, TA813378), and p53 (DO-1, produced in-house) at 1:500 dilution. The secondary antibody used was horseradish peroxidase (HRP) conjugated rabbit anti-mouse (Dako, 1:1000) or HRP-conjugated goat anti-rabbit/mouse IgG (Jackson ImmunoResearch, 1:3000) for Co-IP. Immunoblots were processed by chemiluminescent substrate (Clarity MaxTM Western ECL Substrate, BIO-RAD).

Either Flo-1 or A549 cells were cultured on 10 cm plates and next day the cells were treated with 10 ng/ml IFNα14 for 24 h. Cells were scraped on ice, lysed with the 400 µl of ice-cold lysis buffer (150 mM NaCl, 25 mM Tris–HCl, 0.5% Triton x-100; pH 7.5) with protease inhibitors and centrifuged at 12,000×*g* for 15 min. 140 µg of lysate was used for immunoprecipitation. Lysates were pre-cleared with 5 µl protein G magnetic beads (Thermo Fisher) for 30 min at 4 °C. Further, the pre-cleared lysates were incubated overnight with 3 ug of antibodies against MDN1 (Thermo Fisher, cat. No. A304-739A), histone H2BFS (Thermo Fisher, cat. No. MA5-31410) or unspecific IgG at 4 °C followed by 30 min binding to the Protein G beads (20 µl/sample). Beads were then washed three times in PBS and proteins were eluted in a 2× SDS-loading buffer at 50 °C for 10 min followed by immunoblotting.

## Supplementary Information

Supplementary Information.

Supplementary Tables.

## Data Availability

All data generated or analysed during this study are included in this published article and its Supplementary Information files.
