# Supplementary material for: Identification of novel interferon responsive protein partners of human leukocyte antigen A (HLA-A) using cross-linking mass spectrometry (CLMS) approach
Source: Sci Rep. 2022 Nov 12;12:19422. doi: 10.1038/s41598-022-21393-z (PMC9653400; doi:10.1038/s41598-022-21393-z)
Supplement: Supplementary file 1 — Supplementary Information. [file 41598_2022_21393_MOESM1_ESM.pdf]

Identification of novel interferon responsive protein partners of human leukocyte antigen A (HLA-A) using cross-linking approach

Ashita Singh[1,2], Monikaben Padariya[3], Jakub Faktor[3], Sachin Kote[3], Sara Mikac[3], Alicja Dziadosz[3], Tak W. Lam[1], Jack Brydon[1], Martin A. Wear[5], Kathryn L. Ball[1], Ted Hupp[1,3], Alicja Sznarkowska[3], Borek Vojtesek[4]*, Umesh Kalathiya[3]*

[1] Institute of Genetics and Molecular Medicine, University of Edinburgh, Edinburgh, Scotland EH4 2XR, UK

[2] Department of Experimental Biology, Faculty of Science, Masaryk University, Kamenice 5, 625 00 Brno, Czech Republic

[3] University of Gdansk, International Centre for Cancer Vaccine Science, ul. Kładki 24, 80-822 Gdansk, Poland

[4] RECAMO, Masaryk Memorial Cancer Institute, Zlutykopec 7, 65653 Brno, Czech Republic.

[5] Institute of Structural and Molecular Biology, School of Biological Sciences, University of Edinburgh, Edinburgh EH9 3JR, UK.

*Correspondence: vojtesek@mou.cz (B.V), umesh.kalathiya@ug.edu.pl (U.K)

Supporting Materials

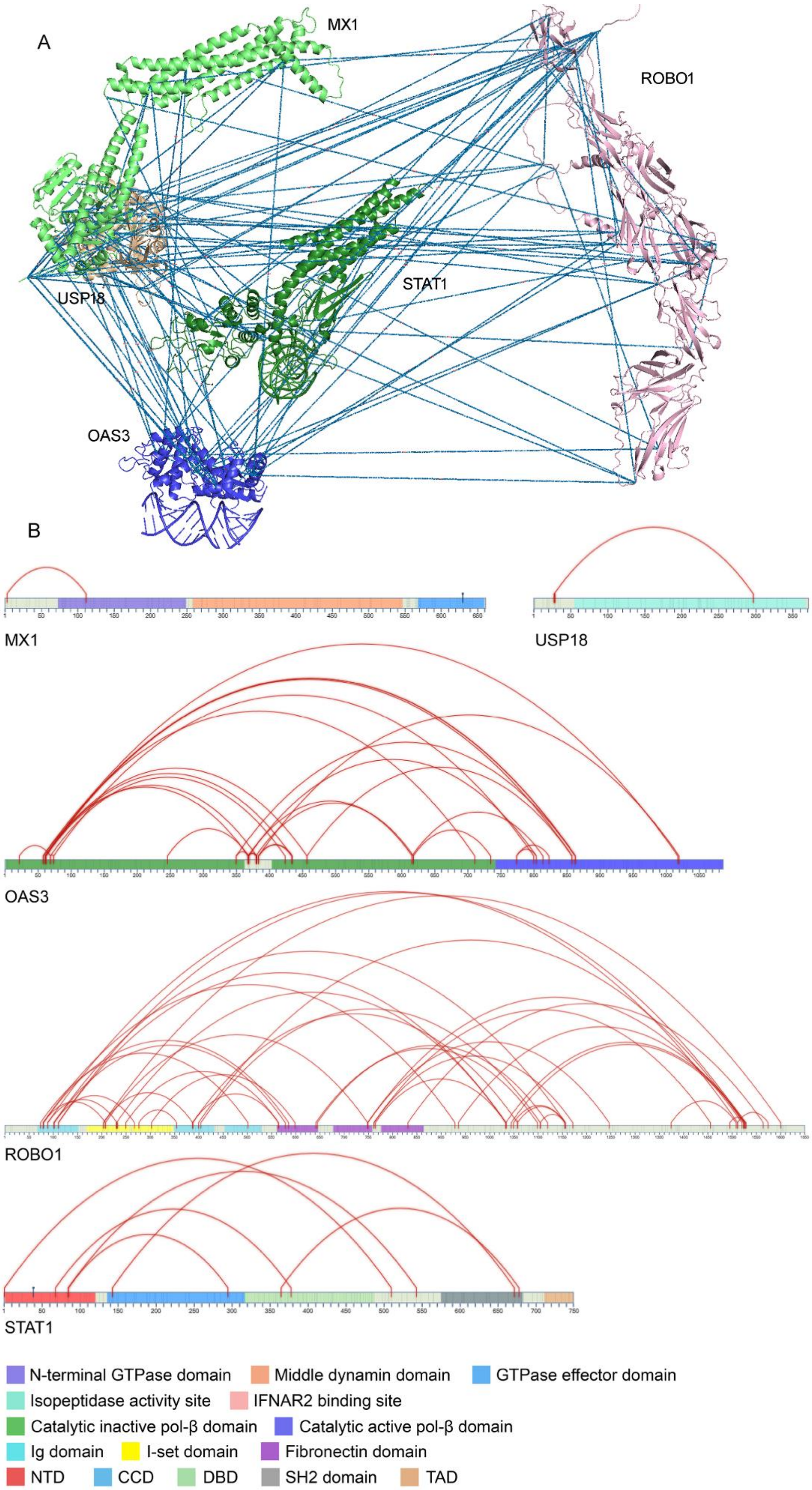

Figure S1. Different interactions represented in Figure 4 (from the main text) between the MX1, UBP18, OAS3, ROBO1, and STAT1 overlaid over their protein structure in PyMol (The PyMOL Molecular Graphics System, Version 2.0 Schrödinger, LLC.). The following structure were retrieved from

AlphaFold (MX1, UBP18, and ROBO1) server, and the crystal structures of STAT1 (pdb id.: 1bf5) and OAS3 (pdb id.: 4s3n) were considered.

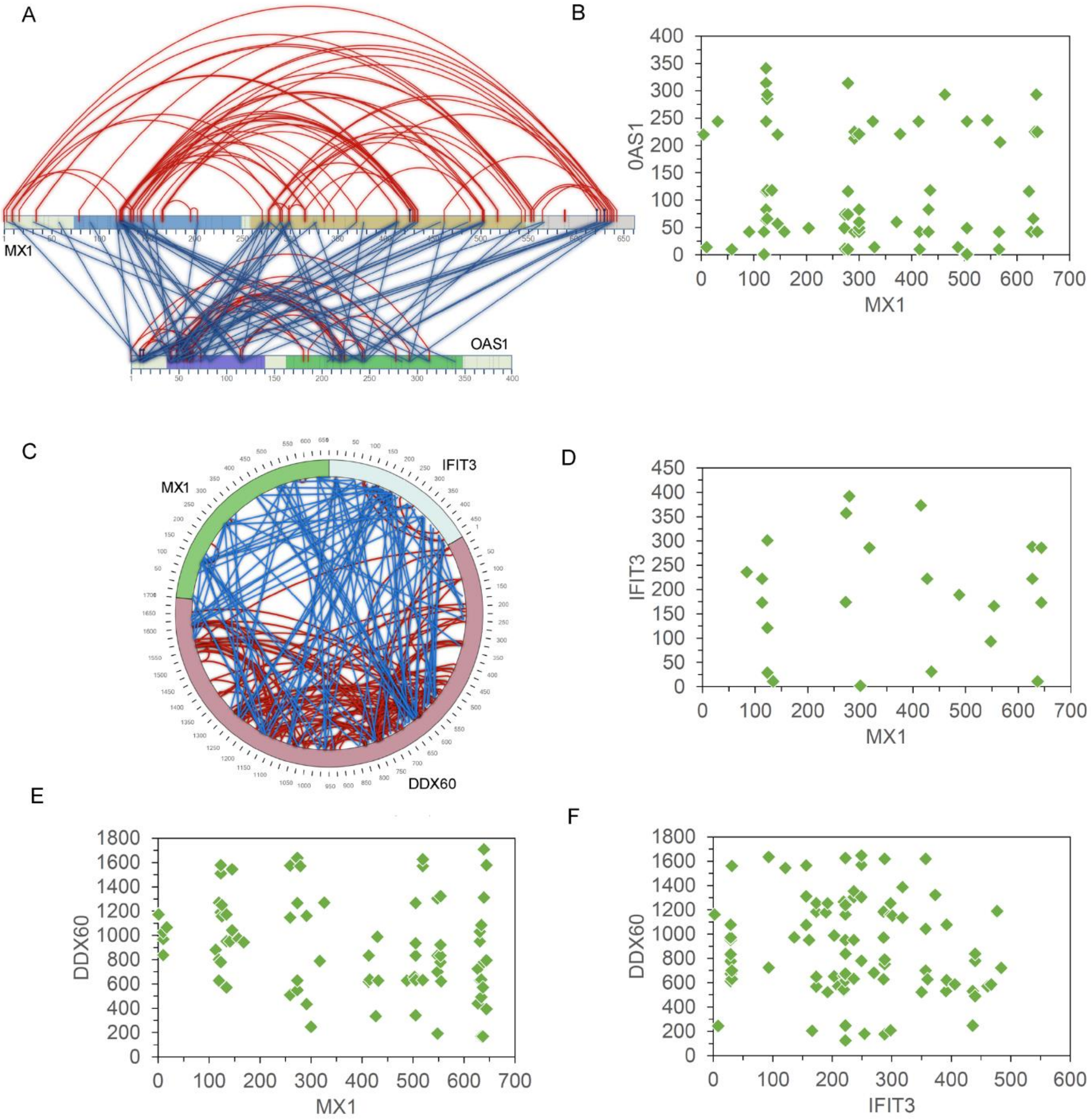

Figure S2. Protein-protein intermolecular network of ISGs identified in the IFNα14 treated cross-linked Flo-1 cells. **(A)** The cross-linked proteins (MX1 and OAS1), with identified inter- and intra- interactions colored in blue and red, respectively. For the right panel the cross-link score cut-off was set to 3.5 and the left panel cut-off was set to 3.0. **(C)** 2D interactive map showing the protein-protein interactions (MX1, IFIT3, and DDX60), the lines represent intermolecular interactions. **(B)**, **(D)**, **(E)**, and **(F)** The plots represent the interacting sites of MX1, OAS1, IFIT3 and DDX60, with labeled interaction site K or S between two peptides. In the plots, the cross-link score cut-off was set to 3.0.

A

```
HLA-B MRVTAPRTLLLLLWGAVALTETWAGSHSMRYFYTAMSRPGRGEPRFITVGYVDDTLFVRF     60
HLA-A XAVMAPRTLVLLLSGALALTQTWAGSHSMRYFFTSVSRPGRGEPRFIAVGYVDDTQFVRF     60
        * *****:*** **:***:***********:*::***********:******* ****
HLA-B DSDATSPRKEPRAPWIEQEGPEYWDRETQISKTNTQTYRENLRTALRYYNQSEAGSHIIQ     120
HLA-A DSDAASQRMEPRAPWIEQEGPEYWDGETRKVKAHSQTHRVDLGTLRGYYNQSEAGSHTVQ     120
      ****:* * **************** **:   *:::**:* :* *   ********** :*
HLA-B RMYGCDVGPDGRLLRGYDQDAYDGKDYIALNEDLRSWTAADTAAQITQRKWEAARVAEQR     180
HLA-A RMYGCDVGSDWRFLRGYHQYAYDGKDYIALKEDLRSWTAADMAAQTTKHKWEAAHVAEQL     180
      ******** * *:****.* **********:********** *** *::*****:****
HLA-B RAYLEGECVEWLRRYLENGKETLQRADPPKTHVTHHPISDHEVTLRCWALGFYPAEITLT     240
HLA-A RAYLEGTCVEWLRRYLENGKETLQRTDAPKTHMTHHAVSDHEATLRCWALSFYPAEITLT     240
      ****** *****************:* ****:***  :****.*******.*********
HLA-B WQRDGEDQTQDTELVETRPAGDRTFQKWAAVVVPSGEEQRYTCHVQHEGLPKPLTLRWEP     300
HLA-A WQRDGEDQTQDTELVETRPAGDGTFQKWAAVVVPSGQEQRYTCHVQHEGLPKPLTLRWEP     300
      ********************** *************:***********************
HLA-B SSQSTVPIVGIVAGLAVLAVVVIGAVVAAVMCRRKSSGGKGGSYSQAACSDSAQGSDVSL     360
HLA-A SSQPTIPIVGIIAGLVLFGAVITGAVVAAVMWRRKSSDRKGGSYSQAASSDSAQGSDVSL     360
      *** *:*****:***.::..*: ******** *****. *********.***********
HLA-B TA--- 362
HLA-A TACKV 365
      **
```

Sequences in red (HLA-A): XAVMAPRTLVLLLSGALALTQTWAGSHSMRYFFTSVSRPGRGEPRFIAVGYVDDTQFVRF; DSDAASQRMEPR; RKVKAHSQTHRVDLGTLRGYYNQSEAGSHTVQ; R; SWTAADMAAQTTKHKWEAAHVAEQL; RAYLEGTCVEWLR; YLENGKETLQRTDAPKTHMTHHAVSDHEATLRCWALSFYPAEITLT; WQR; PAGDGTFQKWAAVVVPSGQEQRYTCHVQHEGLPKPLTLR; RKSSDRKGGSYSQAASSDSAQGSDVSL; TACKV.

B

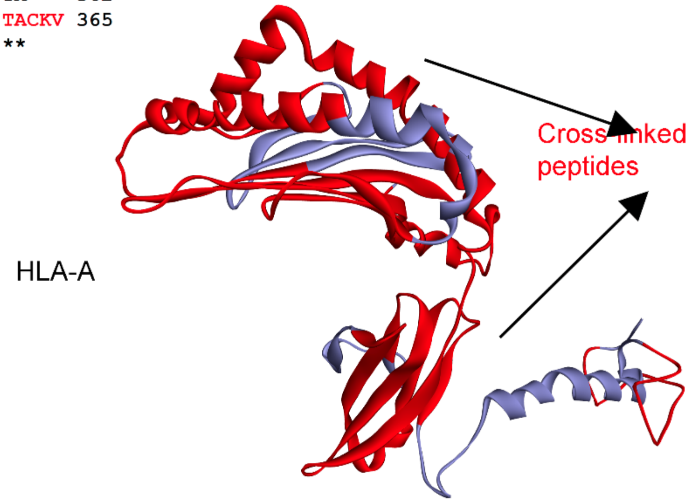

C

```
HLA-C MRVMAPRTLILLLSGALALTETWACSHSMRYFYTAVSRPGRGEPRFIAVGYVDDTQFVQF     60
HLA-A XAVMAPRTLVLLLSGALALTQTWAGSHSMRYFFTSVSRPGRGEPRFIAVGYVDDTQFVRF     60
        *******:**********:*** *******:*:************************:*
HLA-C DSDAASPRGEPRAPWVEQEGPEYWDRETQKYKRQAQTDRVNLRKLRGYYNQSEAGSHTLQ     120
HLA-A DSDAASQRMEPRAPWIEQEGPEYWDGETRKVKAHSQTHRVDLGTLRGYYNQSEAGSHTVQ     120
      ****** * ******:********* **:* * ::**.**:* .**************:*
HLA-C RMYGCDLGPDGRLLRGYNQFAYDGKDYIALNEDLRSWTAADTAAQITQRKWEAARVAEQG     180
HLA-A RMYGCDVGSDWRFLRGYHQYAYDGKDYIALKEDLRSWTAADMAAQTTKHKWEAAHVAEQL     180
      ******:* * *:****:*:**********:********** *** *::*****:****
HLA-C RAYLEGTCVEWLRRYLENGKETLQRAEHPKTHVTHHPVSDHEATLRCWALGFYPAEITLT     240
HLA-A RAYLEGTCVEWLRRYLENGKETLQRTDAPKTHMTHHAVSDHEATLRCWALSFYPAEITLT     240
      *************************:: ****:*** **************.*********
HLA-C WQRDGEDQTQDTELVETRPAGDGTFQKWAAVVVPSGEEQRYTCHVQHEGLPEPLTLRWGP     300
HLA-A WQRDGEDQTQDTELVETRPAGDGTFQKWAAVVVPSGQEQRYTCHVQHEGLPKPLTLRWEP     300
      ************************************:***************:****** *
HLA-C SSQPTIPIVGIVAGLAVLAVLAVLGAVMAVVMCRRKSSGGKGGSCSQAASSNSAQGSDES     360
HLA-A SSQPTIPIVGIIAGLVLFGA-VITGAVVAAVMWRRKSSDRKGGSYSQAASSDSAQGSDVS     359
      ***********:***.::..  .: ***:*.** *****. **** ******:****** *
HLA-C LIACKASNSAQGSDESLIACKA   382
HLA-A LTACKV----------------   365
      * ***.
```

D

Gene	Non-cross-linked		Cross-linked	
	Untreated	IFNα	Untreated	IFNα
HLA-A	79553000	19666000	17476000000	39801000000
HLA-B	6489900	15074000	1776400000	7778900000
HLA-C	4909500	2758000	2196600000	11652000000

Figure S3. Multiple sequence alignment of HLA proteins' amino acid sequences specific to Flo-1 cells. **(A)** Amino acid sequence alignment between HLA-A and -B, and between **(C)** HLA-A and -C. Sequences in red are the cross-linked peptides identified in complex with H2BFS/HMGA1. An *

(asterisk) indicates fully conserved residues: (colon) indicates conservation between groups of strongly similar properties, and a gap indicates dissimilar amino acids. **(B)** A model of Flo-1 specific HLA-A protein using BIOVIA Discovery Studio Visualizer (Dassault Systèmes, BIOVIA) with crosslinked regions highlighted in red. **(D)** A table representing relative intensity of peptides identified for HLA-A, -B and –C in non-cross-linked and cross-linked samples.

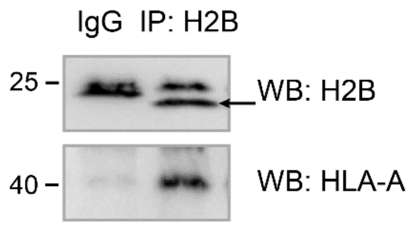

Figure S4. HLA-A co-purifies with H2B in A549 cells. Representative immunoblotting of endogenous H2B immunoprecipitated from A549 cells and probed with the indicated antibodies. Rabbit IgG was used as negative control (IgG).

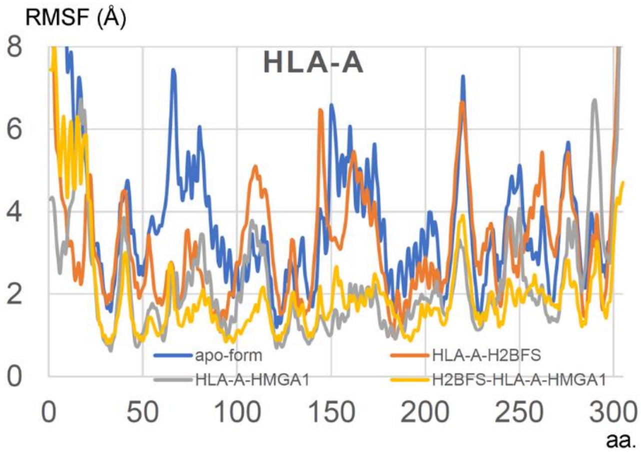

Figure S5. Conformational dynamics of the possible network between H2BFS-HLA-A, HMGA1-HLA-A and H2BFS-HLA-A-HMGA1 complexes. Root-mean-square fluctuations (RMSFs) of individual amino acids within the protein structure (computed based on the C-alpha atoms).

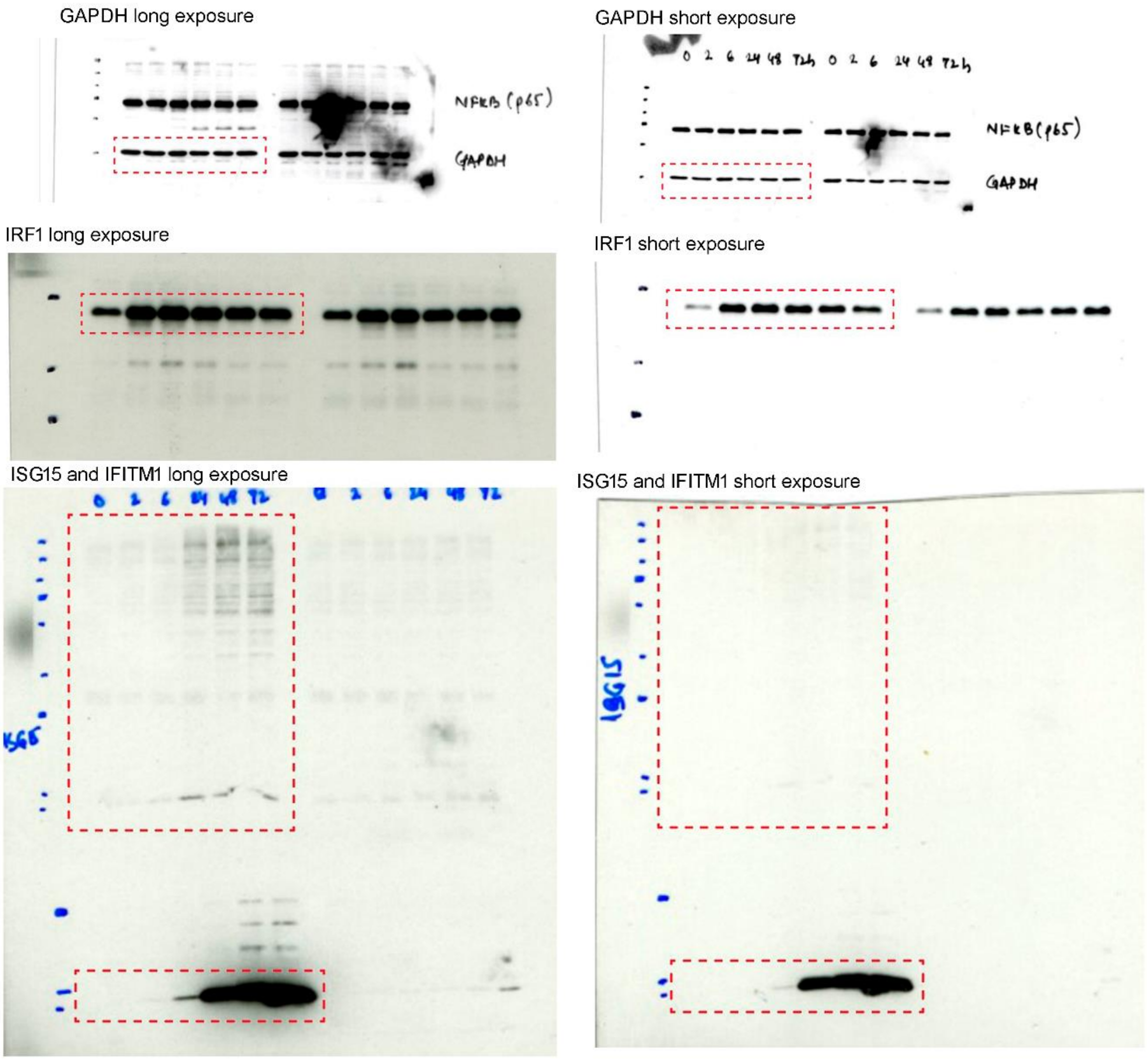

Figure S6 (Part_A). Original blots for the Figure 1. Differential protein expression response in Flo-1 cells following IFNα treatment. Blots for this figure were cut prior to hybridisation with the antibody to avoid running multiple gels.

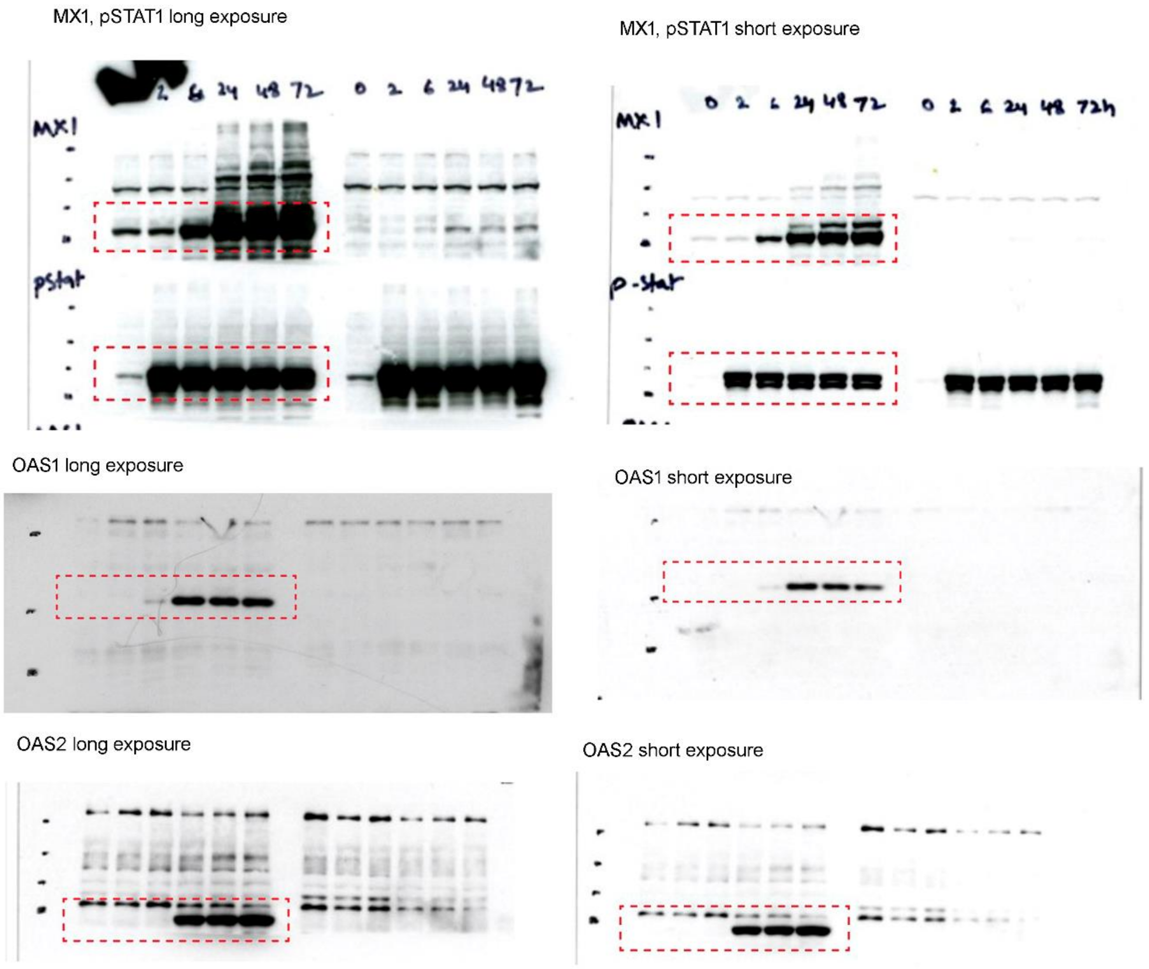

Figure S6 (Part_B). Original blots for the Figure 1. Differential protein expression response in Flo-1 cells following IFNα treatment. Blots for this figure were cut prior to hybridisation with the antibody to avoid running multiple gels.

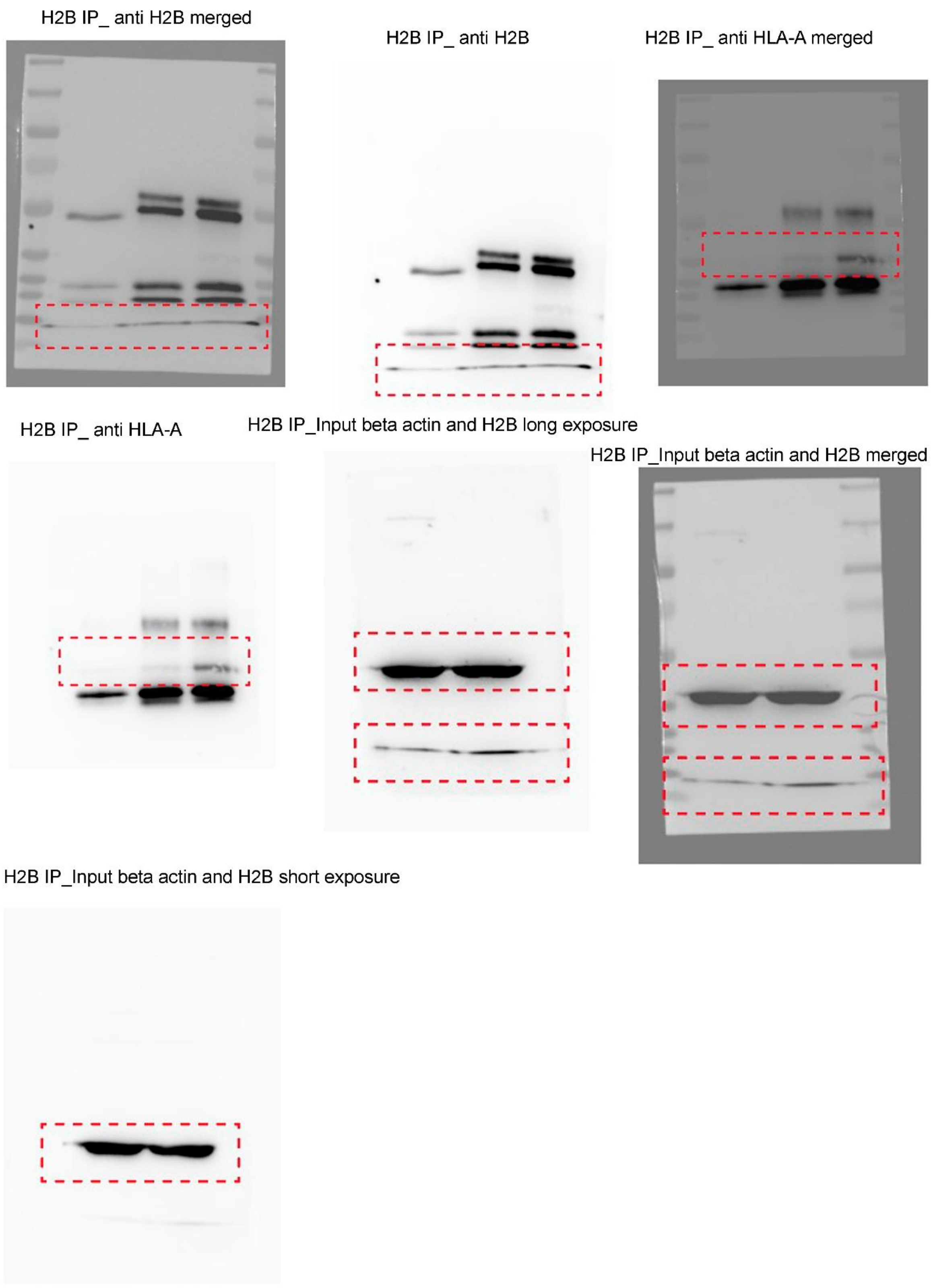

Figure S7 (Part_A). Original blots for the Figure 7. HLA-A co-purifies with H2B and MDN1. Blots were run full-length for this figure.

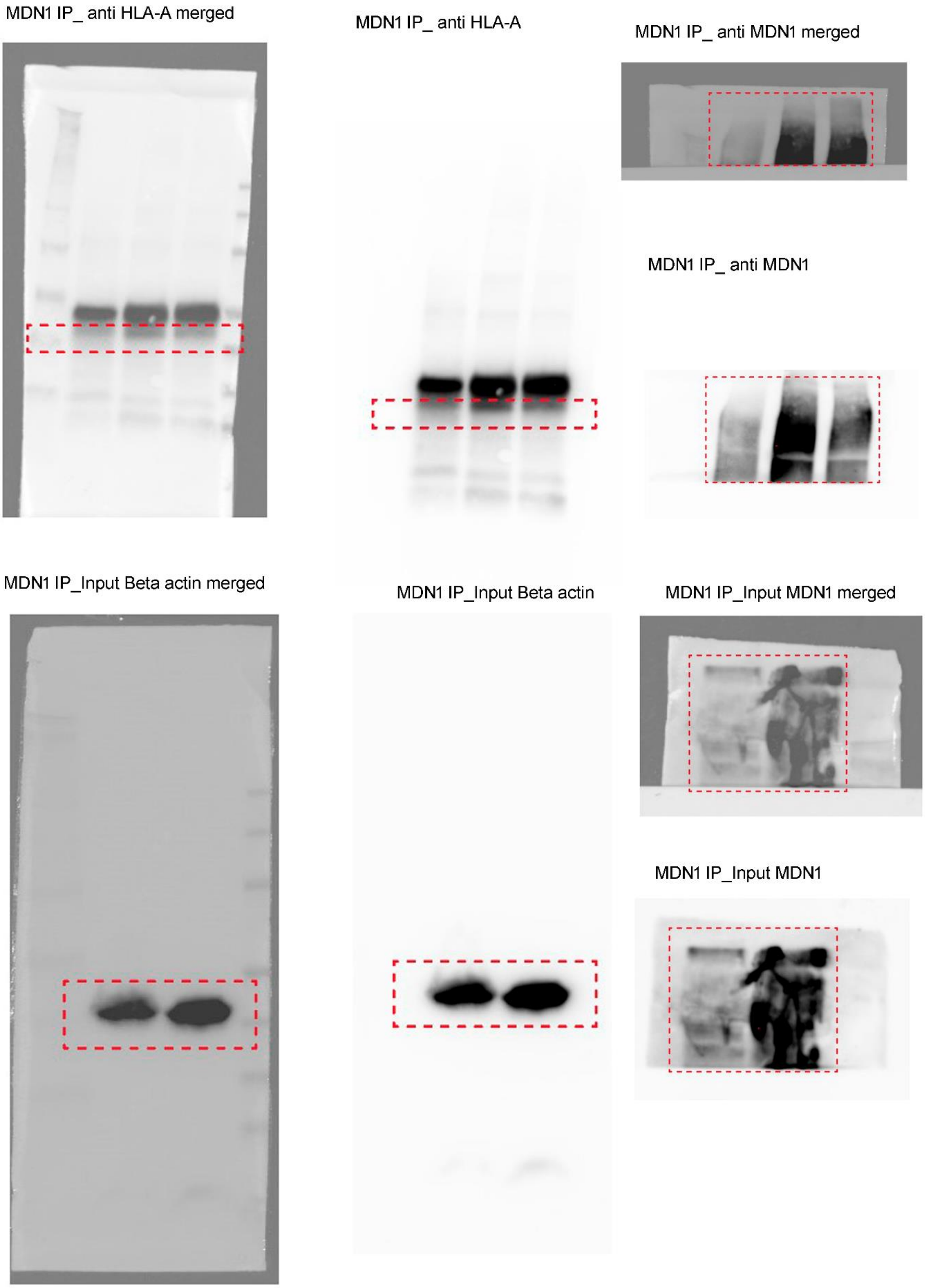

Figure S7 (Part_B). Original blots for the Figure 7. HLA-A co-purifies with H2B and MDN1. Blots were run full-length for this figure.

Table S1. List of proteins identified applying the MaxQuant pipelines, from the cross-linked IFNα14 treated and untreated Flo-1 cells (attached as a separate file).

Table S2. Identified cross-link and the protein intermolecular network of ISGs identified in the IFNα14 treated cross-linked Flo-1 cells for the following protein: MX1, UBP18, OAS3, ROBO1, STAT1, IFIT3, OAS1, and DDX60 (attached as a separate file).

Table S3. Cross-link sites identified or the protein intermolecular network of MHC-I molecules identified in the IFNα14-treated DSS cross-linked Flo-cells for the following protein: H2BFS, HLA-A, MDN1, LRCH4, and HMGA1 (attached as a separate file).

Table S4. A list of proteins and peptides identified in the non-cross-linked IFNα treated and untreated Flo-1 cells. Peptides and proteins were identified and quantified using the MaxQuant package and the statistical analysis was performed using the Perseus program (version 1.6.10.45).

Table S5. Intermolecular interactions between H2BFS-HLA-A-HMGA1 cross-link proteins, identified from MD simulation approach. Long lasting protein-protein intermolecular H-bond interactions ≥ 10 ns are presented in this table.

**HLA-A-H2BFS**			**HLA-A-HMGA1**			**H2BFS-HLA-A-HMGA1**					
**HLA-A**	**H2BFS**	**Occup.**	**HLA-A**	**HMGA1**	**Occup.**	**HLA-A**	**H2BFS**	**Occup.**	**HLA-A**	**HMGA1**	**Occup.**
Glu201	Arg34	60.68%	Asp61	Arg28	91.52%	Arg193	Asp26	86.23%	Asp61	Arg28	87.43%
Arg205	Glu114	50.70%	Arg258	Glu22	60.18%	Glu197	Arg34	67.07%	Asp220	Arg73	73.35%
Glu82	Arg32	39.52%	Asp262	Arg30	37.03%	Arg205	Glu114	57.09%	Asp262	Arg24	65.77%
Arg193	Glu3	33.13%	Glu222	Arg73	32.83%	Phe133	Lys24	49.00%	Val218	Lys74	58.18%
Glu82	Arg28	30.14%	Glu256	Lys65	22.46%	Glu82	Arg32	47.60%	Arg226	Glu104	57.09%
Glu197	Met1	25.75%	Glu253	Arg81	20.86%	Glu185	Arg28	47.41%	Leu254	Gln100	56.39%
Glu288	Lys121	23.25%	Glu256	Lys31	16.37%	Asp207	Lys44	40.92%	Leu254	Arg60	46.31%
Lys210	Asp52	22.65%	Gln120	Thr39	16.27%	Glu185	Gln23	36.13%	Asp251	Arg58	46.31%
Pro74	Tyr43	22.46%	Gln120	Gly38	15.57%	Arg258	Asp52	33.13%	Glu253	Arg60	43.41%
Glu197	Lys31	21.56%	Thr224	Ala70	13.77%	Glu185	Lys21	22.06%	Thr252	Gln100	33.03%
Arg193	Asp26	19.56%	Gln120	Ser36	11.18%	Arg181	Gln23	16.77%	Glu256	Arg30	18.56%
Gln78	Lys35	18.26%	Arg41	Glu17	10.58%	Asn64	Gln78	14.87%	Asp220	Thr75	16.97%
Asp207	Lys44	16.77%				Glu190	Lys29	14.67%	Asp262	Arg28	12.57%
Glu79	Lys31	15.87%				Thr264	Asp52	13.47%			
Arg205	Tyr41	13.77%				Lys35	Gln78	12.38%			
Asp207	Lys47	12.28%				Glu197	Lys31	11.88%			
Asp262	Ile55	11.68%				Glu178	Lys17	10.18%			
Thr206	Tyr43	11.08%									
Glu190	Lys29	10.48%									
